# Research Progress on Chemical Components of *Astragalus membranaceus* and Treatment of Metabolic Syndrome

**DOI:** 10.3390/molecules30183721

**Published:** 2025-09-12

**Authors:** Taiyu Liu, Yumu Sun, Xueying Zhao

**Affiliations:** School of Basic Medical Sciences, Heilongjiang University of Chinese Medicine, 24 Heping Road, Harbin 150040, China; 13946539517@163.com (T.L.); 17703607675@163.com (Y.S.)

**Keywords:** *Astragalus membranaceus*, chemical constituents, metabolic syndrome, type 2 diabetes mellitus, non-alcoholic fatty liver disease, obesity, hypertension, cardiovascular diseases

## Abstract

*Astragalus membranaceus* (AM), also known as Huangqi in Chinese, refers to the dried root of two Leguminosae species: AM (Fisch.) Bge. and its variety AM (Fisch.) Bge. var. *mongholicus* (Bge.) Hsiao. In recent years, research on AM has been published in many papers. Its role in treating metabolic syndrome (MetS) has attracted increasing attention. This review summarizes the research progress over the past five years on the chemical constituents of AM and its therapeutic potential in MetS-related diseases. Chemical analyses of AM have gradually expanded from its roots to its stems, leaves, and entire plant. The major compounds isolated from AM include flavonoids, saponins, and polysaccharides. Extraction methods include ultra-performance liquid chromatography coupled with tandem mass spectrometry and in vitro intestinal absorption models combined with high-performance liquid chromatography–photodiode array–tandem mass spectrometry. AM and its active components exert beneficial effects on metabolic disorders such as type 2 diabetes mellitus, non-alcoholic fatty liver disease, obesity, hypertension, and cardiovascular diseases. These effects are achieved mainly through mechanisms such as reducing oxidative stress and inflammation, modulating gut microbiota, regulating lipid metabolism, improving insulin resistance, and protecting pancreatic β-cell function. This review provides a reference for further studies on treatment of MetS of AM.

## 1. Introduction

*Astragalus membranaceus* (AM), also known as Huangqi in Chinese, is the dried root of *Astragalus membranaceus* (Fisch.) Bge. var. *mongholicus* (Bge.) Hsiao or *Astragalus membranaceus* (Fisch.) Bge., which belongs to the Leguminosae family. AM has a mild aroma and a slightly sweet taste. When chewed, it produces a faint beany flavor. In traditional Chinese medicine theory, AM can enter the lung and spleen meridians and is traditionally used to tonify Qi, raise Yang, consolidate the exterior to stop sweating, promote urination to reduce swelling, generate body fluids, nourish the blood, dispel stasis, discharge pus, and promote wound healing [1].

Over recent decades, AM has been extensively studied in terms of its chemical constituents, pharmacological effects, and therapeutic indications. For instance, Tian et al. provided a comprehensive overview of the extraction, structural characteristics, biological activities, and practical applications of astragalus polysaccharides (APSs) isolated from AM [2]. APSs demonstrated a wide range of biological functions, including immunomodulatory effects, anti-tumor activity, anti-diabetic properties, and cardiovascular protective effects. Nevertheless, further investigation is required to elucidate their purification methods, structure–activity relationships, and underlying mechanisms of action [2]. Some research on AM in immune regulation has been published [3,4,5]. The authors reported that AM can promote the development of immune organs, enhance mucosal immune function, improve innate immune phagocytic capacity, promote the maturation and differentiation of acquired immune cells, and improve antibody expression. In addition, AM has a broad research space in the adjuvant treatment of immune-related diseases. Based on the above functions, AM shows significant development value in the treatment of cancer, autoimmune diseases such as multiple sclerosis, type 1 diabetes, rheumatoid arthritis, etc. Furthermore, AM has excellent anti-tumor activity by inducing apoptosis of cancer cells, inhibiting tumor angiogenesis, regulating the cell cycle and microenvironment, and reversing tumor drug resistance [6,7,8,9,10,11]. AM can also alleviate the inflammatory cascade reaction in allergic rhinitis and asthma. This effect is mainly mediated through key mechanisms: inhibiting the nuclear factor kappa B (NF-κB) pathway, correcting the imbalance between T helper 17 (Th17) cells and regulatory T (Treg) cells, and blocking histamine release [12,13,14]. Additionally, AM has good anti-inflammatory and antiviral effects and can be used to treat inflammatory bowel disease [15,16]. In summary, AM has good therapeutic effects against cancer cells, allergic reactions, and inflammatory responses.

Several studies have also documented the metabolic regulatory effects of AM. Zhang et al. reviewed multiple pathways through which APSs improved non-alcoholic fatty liver disease (NAFLD) and pointed out its role in regulating the intestinal flora [17]. Liu et al. emphasized the molecular mechanisms by which APSs improved diabetic complications [18]. Chen et al. summarized the mechanism by which astragaloside IV (AsIV) can treat various liver diseases [19]. Additionally, AsIV exerts a favorable protective effect on the heart. It can inhibit myocardial hypertrophy and fibrosis, enhance cardiac function, improve vascular endothelial dysfunction, promote angiogenesis, and regulate key signaling pathway targets associated with cardiovascular diseases (CVDs) [20,21].

In short, most of the reported reviews focus on a single disease or organ, without summarizing the multi-target mechanisms within the overall framework of metabolic syndrome (MetS) or involving the core pathways of MetS. Focusing on a certain type of component while ignoring the synergistic effects among different components.

In recent years, research has extended beyond the root to explore the medicinal potential of other plant parts, such as stems and leaves. As a traditional Chinese medicinal herb with multi-component and multi-target properties, AM shows advantages in the management of MetS. Currently, the therapeutic value of AM in MetS and its component diseases has not been systematically organized. Similarly, the specific mechanisms underlying its actions in these conditions remain to be deeply discussed. The majority of published studies have primarily focused on MetS-related risk factors associated with AM, such as diabetes, complications, and obesity, while failing to explore the common pathological basis and distinct pathogenic mechanisms underlying these conditions. To further investigate the therapeutic potential of AM, we conducted a systematic review of recent original research articles focusing on its application in managing MetS and associated risk factors. A literature search was conducted across PubMed, ScienceDirect, Web of Science, and CNKI (China National Knowledge Infrastructure) using the keywords “*Astragalus membranaceus*”, “huangqi” “metabolism”, and “metabolic syndrome” to ensure broad coverage of both English- and Chinese-language publications. The inclusion criteria were defined as follows: AM and its active components in the context of MetS, including related conditions such as diabetes, NAFLD, obesity, hypertension, cardiovascular diseases, as well as pharmacological investigations of AM. Studies that were duplicated or incomplete, lacked ethical approval, or were classified as conference abstracts or brief communications were excluded.

## 2. Main Components of AM

With the advancement of isolation and identification techniques, research on the chemical constituents of AM has become increasingly detailed. The investigation of active ingredients in Chinese medicine now extends beyond phytochemical methods. Screening for bioactive compounds is essential to elucidate the material basis of pharmacological efficacy [22]. Due to the limited availability of wild AM resources, its commercial development is restricted. Recent studies have focused on the utilization of stems and leaves besides the root, the development of AM from alternative geographical sources, and the characterization of key compounds affected by origin [23,24,25].

Some researchers employed in vitro intestinal absorption models combined with high-performance liquid chromatography–photodiode array–tandem mass spectrometry (HPLC-PDA-MS) to rapidly screen and analyze potential active compounds in multi-component systems. A response surface methodology was used to optimize extraction and separation conditions [22,26,27]. Zhao et al. [28] established chromatographic fingerprints of AM from different regions using the HPLC-Evaporative Light Scattering Detector (ELSD) method. They found that AM from Inner Mongolia of China most closely resembled the wild variety. This method also provides a reference for the conservation and substitution of wild AM resources, as well as for cultivation and processing [28].

In this paper, we summarized many studies from the past five years on newly isolated and identified compounds and key active constituents from AM including flavonoids, saponins, and polysaccharides.

### 2.1. Astragalus Flavonoids

**Figure 1:**
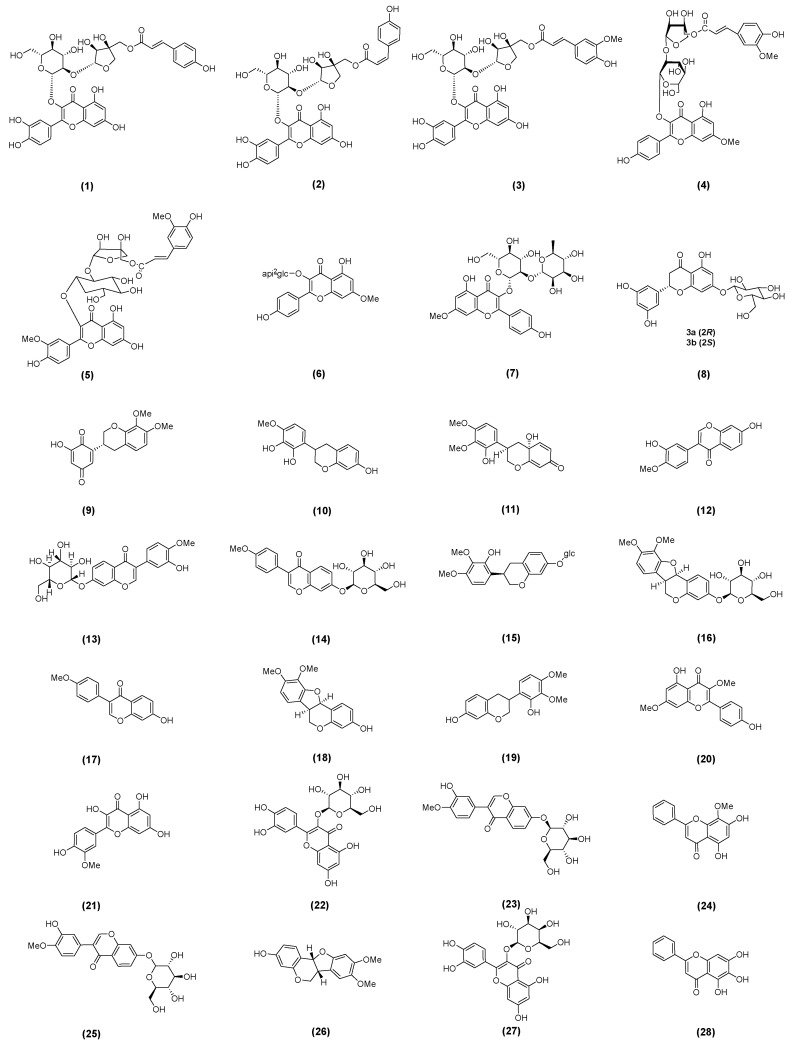
Some flavonoids isolated from AM (Numbers (**1**)–(**28**) represent compound numbers).

Flavonoids are the largest group of plant secondary metabolites and share a common C6-C3-C6 backbone [29]. Recently, some flavonoids were isolated and identified from AM, and their corresponding structures are shown in Figure 1. Tan et al. [30] successfully isolated and identified three novel flavonoid glycosides and eight known analogs from the stems and leaves of AM. These include astragaloside A (**1**), B (**2**), C (**3**), 3-O-[5‴-O-feruloyl-β-D-apiofuranosyl(1‴→2″)-β-D-glucopyranosyl] rhamnocitrin (**4**), isorhamnetin 3-O-[5-O-trans- feruloyl-β-D-apiofuranosyl-(1→2)-β-D-glucopyranoside (**5**), rhamnocitrin 3-O-β-D-apiofuranosyl(1→2)-β-D-gluco-pyrano-side (**6**), rhamnocitrin-3-O-β-neohesperidoside (**7**), 5,3′,5′-trihydroxy-flavanone-7-O-β-D-glucopyranoside (**8**), (3R)-astragaluquinone (**9**), arizonicanol A (**10**), and astragaisoflavan A (**11**) [30]. Wang et al. [31] analyzed 19 batches of AM from different zones of China. The content of chemical components in the stems and leaves varied significantly with harvest time and plant age [31]. Li et al. used HPLC–ultraviolet (UV)/ELSD to compare the active constituents of AM and investigated the effects of different varieties and active compounds on nephrotic syndrome (NS), including calycosin (**12**), calycosin-7-O-β-D-glucoside (**13**), ononin (**14**), isomucronulatol-7-O-β-D-glucoside (**15**), methylnissolin-3-O-glucoside (**16**), formononetin (**17**), methylnissolin (**18**), and isomucronulatol (**19**) [32].

Wang et al. identified potential active compounds such as kumatakenin (**20**), isorhamnetin (**21**), isoquercitrin (**22**), and calycosin-7-O-glucopyranoside (**23**) by combining an in vitro intestinal absorption model with HPLC-PDA-MS [22]. Liu et al. [27] developed a receptor–ligand affinity ultrafiltration–liquid chromatography method for active compound separation in the stems and leaves of AM. The identified flavonoids included wogonin (**24**), calycosin-7-glucoside (**25**), 3-hydroxy- 9,10-dimethoxyptercarpan (**26**), hyperoside (**27**), and baicalein (**28**) [27].

### 2.2. Astragalus Saponins

**Figure 2:**
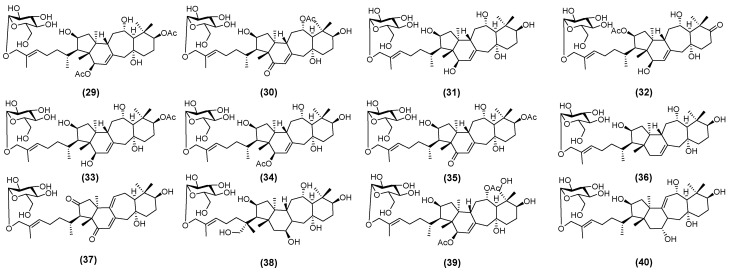
Some saponin compounds isolated from the leaves of AM (Numbers (**29**)–(**40**) represent compound numbers).

Studies indicate that the saponin types in AM leaves are similar to those in roots. However, the total saponin content in leaf is higher than that in root [33]. Yan et al. [34] isolated and identified twelve triterpenoid saponins from AM leaves. These saponins are illustrated in Figure 2 and named huangqiyenins M (**29**), N (**30**), O (**31**), P (**32**), Q (**33**), R (**34**), S (**35**), T (**36**), U (**37**), V (**38**), W (**39**), and X (**40**) [34].

**Figure 3:**
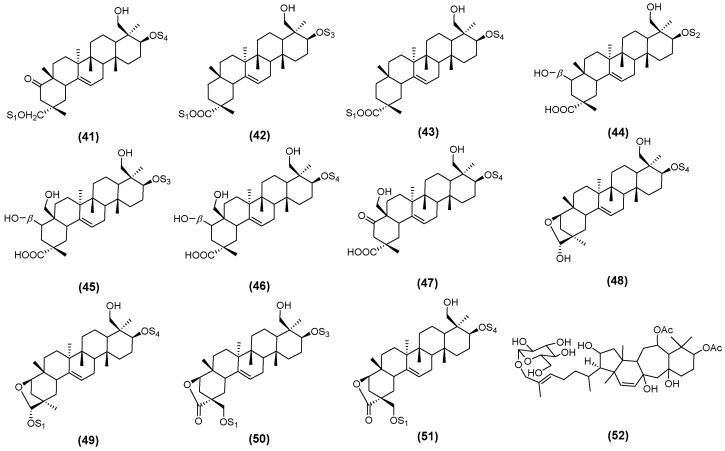
Some saponin compounds isolated from the aerial parts of AM (Numbers (**41**)–(**52**) represent compound numbers).

Similarly, Nguyen et al. [35] isolated eleven novel triterpenoid saponins from the aerial parts of AM. These were designated as astraoleanosides E (**41**), F (**42**), G (**43**), H (**44**), I (**45**), K (**46**), L (**47**), M (**48**), N (**49**), O (**50**), and P (**51**), as illustrated in Figure 3 [35]. Du et al. established a simple and highly sensitive ultra-high-performance liquid chromatography (UHPLC)-MS/MS method to successfully identify huangqiyenin I (**52**) (Figure 3) [36].

**Figure 4:**
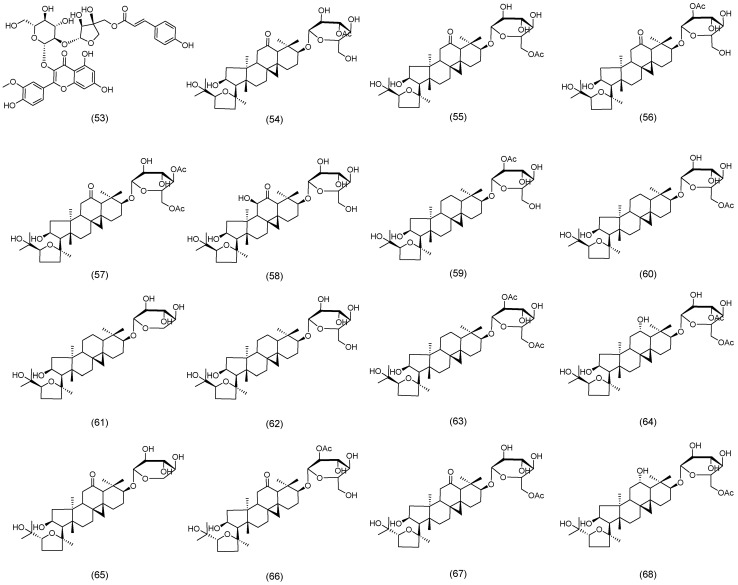
Some saponin compounds isolated from the leaves of AM (Numbers (**53**)–(**68**) represent compound numbers).

**Figure 5:**
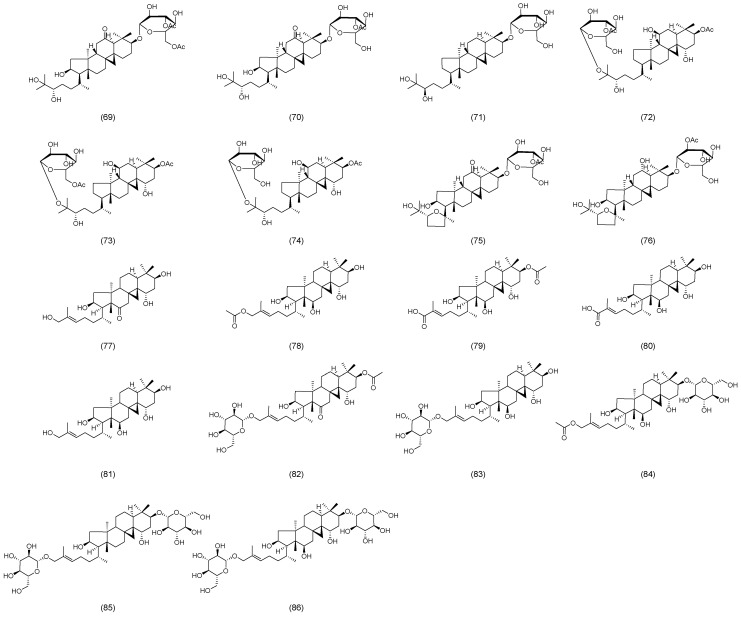
Some saponin compounds isolated from the stems and leaves of AM (Numbers (**69**)–(**86**) represent compound numbers).

Recently, Zhao et al. isolated and identified a new flavonol glycoside, astraside D (**53**), from the stems and leaves of AM, as illustrated in Figure 4 [37]. Xu et al. isolated and characterized fifteen novel triterpene saponins from the leaves of AM, which were designated as huangqiyesaponin A (**54**), B (**55**), C (**56**), D (**57**), E (**58**), F (**59**), G (**60**), H (**61**), I (**62**), J (**63**), K (**64**), L (**65**), M (**66**), N (**67**), and O (**68**) (Figure 4) [38]. In a subsequent study, Xu et al. further identified eight additional new triterpene saponins from the same plant source, and these compounds were named huangqiyesaponin IV (**69**), V (**70**), VI (**71**), VII (**72**), VIII (**73**), IX (**74**), X (**75**), and XI (**76**), as illustrated in Figure 5 [39]. Cheng et al. isolated ten previously unidentified triterpenoids from the stems and leaves of AM, including Cyclastraine A (**77**), B (**78**), C (**79**), D (**80**), E (**81**), F (**82**), G (**83**), H (**84**), I (**85**), and J (**86**) (Figure 5) [40].

### 2.3. Astragalus Polysaccharides

APSs are key bioactive components of AM and mainly consist of heteropolysaccharides and glucans. APSs can be extracted in various ways, such as homogenization-assisted negative-pressure cavitation, juicing extraction and microwave-assisted acid hydrolysis, etc. Enzyme-assisted extraction gives the highest crude polysaccharide yield [41]. Guo et al. [42] developed a UHPLC method with UV detection to specifically identify seven monosaccharide constituents in APSs. These include mannose (Man), rhamnose (Rha), glucose (Glu), galactose (Gal), arabinose (Ara), xylose (Xyl), and fucose (Fuc) [42]. In order to determine the active components of APSs and reduce the risk of clinical adverse reactions, Li et al. [43] classified them into APS-I (>500 kDa) and APS-II (10 kDa) according to molecular weight. Their structures were analyzed by techniques such as methylation analysis (MA), Fourier transform infrared spectroscopy (FT-IR) and nuclear magnetic resonance spectroscopy (NMR), and it was proved that the small molecule APS-II was the main active component of APSs [43]. Chen et al. isolated and purified two homogeneous polysaccharides, APS-A1 (2.62 × 106 Da) and APS-B1 (4.95 × 106 Da), from AM using diethylaminoethyl cellulose column (Type 52) and Sephadex G-100 column [44]. The small molecule components of APSs, such as APS-II, can exert immunomodulatory effects, while the large molecule components, such as APS-A1 and APS-B1, have prominent anti-inflammatory activities. The active characteristics may be related to the specificity of receptor binding. The systematic comparison of the activity differences of APSs in different molecular weight intervals still requires further research.

Currently, studies have shown that the stems and leaves of AM contain abundant flavonoids and saponins. However, whether these components can serve as an effective substitute for the roots in terms of medicinal use and therapeutic outcomes remains unclear. This uncertainty requires systematic evaluation through comparative pharmacodynamic research. The newly isolated compounds, such as astraoleanosides E–P, have so far only undergone structural characterization. To date, no pharmacological evaluation has been conducted on these compounds.

Their mechanisms of action in metabolic regulation remain to be elucidated. Although previous studies have indicated that the pharmacological effects of APSs are influenced by its molecular weight, the specific molecular targets associated with different molecular weight fractions in metabolic regulation remain poorly understood. Future studies could employ metabolomics integrated with animal models. This approach can be used to systematically compare the pharmacological effects of analogous compounds across different plant parts, thereby providing a scientific basis for resource expansion. For newly identified compounds, initial pharmacological screening can be conducted using in vitro cell models, followed by molecular docking to predict potential specific targets. Fractionation purification techniques can be applied to separate APSs into distinct molecular weight fractions, enabling the identification of specific binding receptors. These data can then be compiled into a database to establish clear structure–activity relationships.

## 3. Therapeutic Effects of AM and Its Active Ingredients on MetS

Recent studies demonstrate that AM and its bioactive compounds exhibit significant efficacy in modulating key risk factors of MetS. In this section, the pharmacological actions and pathways were summarized in the treatment of MetS by AM and its components.

### 3.1. Common Mechanisms of AM in MetS

Studies have demonstrated that AM and its components exhibit pharmacological effects including anti-inflammatory and antioxidant activities, modulation of intestinal microbiota, and regulation of lipid metabolism. These effects lay the foundation for the potential of AM in treating kidney diseases, such as acute kidney injury, diabetic nephropathy, hypertensive renal damage, and chronic glomerulonephritis [45,46,47]. AM exerts antioxidant, anti-aging, and metabolic regulatory effects through modulation of key signaling pathways such as general control non-derepressible 2/thioredoxin (GCN2/TXN) and sirtuin 1/tumor protein 53 (SIRT1/p53) [48,49,50,51,52,53]. Furthermore, AM and its active constituents showed improved renal function in patients with diabetic nephropathy. This improvement is evidenced by reductions in proteinuria, serum creatinine, and blood urea nitrogen levels, as well as an increase in glomerular filtration rate [54,55,56,57]. Given its multi-target anti-inflammatory and antioxidant properties, AM demonstrates significant therapeutic potential for MetS-associated diseases.

#### 3.1.1. Antioxidation

Oxidative stress refers to a state of disequilibrium between oxidants and antioxidants during the body’s metabolic processes. This state arises when reactive oxygen species (ROS) and other oxidative agents are overproduced, or when the antioxidant defense system becomes impaired. It serves as a key pathophysiological mechanism contributing to MetS and its associated complications.

Zou et al. [58] demonstrated that AsIV was capable of binding to glycogen synthase kinase-3beta (GSK-3β) through molecular docking, thereby enhancing its phosphorylation. Furthermore, AsIV was shown to upregulate the expression of nuclear factor erythroid 2-related factor 2 (Nrf2) and its downstream antioxidant effectors, heme oxygenase-1 (HO-1) and nicotinamide adenine dinucleotide phosphate (NADPH): quinone oxidoreductase 1 [58]. AsIV-pretreated endothelial progenitor cells effectively alleviated oxidative stress and endothelial dysfunction induced by high glucose (HG) through the *microRNA-210* (*miR-210*)/NADPH oxidase 2/ROS pathway [59]. Zhang et al. [60] demonstrated that AsIV inhibited endothelial dysfunction in the aortas of streptozotocin (STZ)-induced diabetic mice. This effect was associated with reduced NADPH oxidase expression, decreased ROS production, and increased nitric oxide (NO) production [60]. AsIV restores NO production by upregulating the phosphorylation of endothelial nitric oxide synthase (eNOS), thereby improving endothelium-dependent vasodilation. Furthermore, it suppresses the expression of NADPH oxidase subunits, reduces the generation of ROS, and alleviates oxidative stress. Leng et al. [61] indicated that AsIV ameliorated HG-induced endothelial dysfunction by inhibiting the P2X purinoceptor 7 receptor (P2X7R)-dependent p38 mitogen-activated protein kinase (p38 MAPK) pathway both in vivo and in vitro. This resulted in increased eNOS and NO levels, which reduced inflammatory responses and oxidative stress in diabetic model rats [61]. HG activates P2X7R, thereby triggering the p38 MAPK signaling pathway, which results in the release of inflammatory cytokines, increased oxidative stress, and suppression of the eNOS/NO system. AsIV interferes with this pathological cascade by downregulating P2X7R expression and inhibiting p38 phosphorylation.

Liang et al. [62] showed that AsIV reduced liver steatosis, apoptosis, and inflammation in NAFLD. The mechanisms underlying this effect include alleviating oxidative stress, restoring glutathione peroxidase (GSH-Px) levels, suppressing inflammatory cytokine secretion, and downregulating the expression of 5-lipoxygenase (5-LO) and leukotriene A4 hydrolase (LTA4H) [62]. Yuan et al. [63] found that APSs enhanced the body’s antioxidant capacity and mitigated oxidative damage. APSs significantly reduced serum malondialdehyde (MDA) levels and suppressed the accumulation of lipid peroxidation products. Additionally, APSs increased total superoxide dismutase (T-SOD) activity, thereby enhancing the body’s ability to scavenge oxygen free radicals and alleviating oxidative stress-induced injury in liver cells [63].

AsIV reduces the proportion of NADPH oxidase 4 (NOX4)-positive cells in mouse hearts, where NOX4 serves as a key enzyme in ROS production. Furthermore, AsIV enhances the expression of superoxide dismutase 2 (SOD2), a critical antioxidant enzyme that scavenges ROS and mitigates oxidative stress-induced damage in cardiomyocytes [64]. The core mechanism through which total flavonoids of astragalus (TFAs) exert their antioxidant activity involves the direct scavenging of free radicals and the inhibition of free radical generation during ischemia–reperfusion in vivo [65]. AsIV inhibits oxidized low-density lipoprotein (ox-LDL)-induced endoplasmic reticulum stress (ERS) in macrophages. It suppresses the excessive activation of ERS through multiple mechanisms reducing the phosphorylation of inositol-requiring enzyme 1 (IRE1), inhibiting the activating transcription factor 6 (ATF6), and downregulating the expression of glucose-regulated protein 78 (GRP78) and CCAAT/enhancer-binding protein (C/EBP) homologous protein (CHOP). Moreover, AsIV also attenuates ROS-associated ERS. These findings indicate that AsIV may alleviate ERS-mediated cellular damage by either reducing ROS production or scavenging existing ROS [66]. Luo et al. [67] found that AsIV treatment significantly improved cardiac function in rats treated with adriamycin. It regulated autophagy and oxidative stress, reducing heart damage caused by adriamycin through the activation of the phosphatidylinositol 3-kinase (PI3K)/protein kinase B (Akt) pathway [67]. APSs enhanced cholesterol efflux by upregulating the adenosine triphosphate (ATP)-binding cassette transporter A1 (ABCA1), inhibited ox-LDL-induced lipid accumulation in macrophages, regulated lipid levels, and improved the stability of vulnerable plaques [68].

Chan et al. [69] assessed the effect of AM-assisted therapy on renal function decline in patients with type 2 diabetes mellitus (T2DM) complicated by chronic kidney disease (CKD). Patients in the intervention group received AM granules in addition to standard treatment, while those in the control group received only standard treatment. Results showed that patients treated with AM had a significantly slower decline in estimated glomerular filtration rate (eGFR) and a higher eGFR endpoint than the standard care group. Moreover, 48 weeks of AM administration further stabilized renal function compared with standard care alone [69].

AM primarily exerts its effects through multiple mechanisms, including the regulation of redox-related signaling pathways, enhancement of antioxidant enzyme activity, reduction in oxidative substance production, and scavenging of free radicals. However, current mechanistic studies remain limited in depth. There is a lack of systematic investigation into the upstream and downstream molecular targets of individual components and the crosstalk between different signaling pathways.

#### 3.1.2. Anti-Inflammatory

MetS and its associated disorders are characterized by chronic low-grade inflammation. The anti-inflammatory effects of AM are central to mediating its therapeutic actions.

Inflammatory cytokines can impair insulin signaling, accelerate pancreatic β-cell apoptosis, and contribute to vascular complications. AsIV upregulates C1q/tumor necrosis factor-related protein 3 (CTRP3), activates the downstream PI3K/AKT signaling pathway, and results in the downregulation of pro-inflammatory cytokines interleukin-6 (IL-6) and tumor necrosis factor-alpha (TNF-α) [70]. Hoo et al. [71] discovered that the active components extracted from AM included calycosin-7-β-D-glucoside (0.9%), ononin (1.2%), calycosin (4.53%), and formononetin (1.1%). AM can significantly reduce the secretion of pro-inflammatory cytokines TNF-α, IL-6, and monocyte chemoattractant protein-1 (MCP-1) in human Tokyo Hospital Pathology-1 (THP-1) macrophages. They also suppressed lipopolysaccharide (LPS)-induced NF-κB activation in the murine macrophage cell line (RAW264.7) in a dose-dependent manner. This anti-inflammatory activity ameliorated glucose intolerance, insulin resistance (IR), and hypertriglyceridemia in db/db diabetic mice [71]. Zhou et al. [72] reported that AsIV significantly downregulated toll-like receptor 4 (TLR4) and phosphorylated NF-κB subunit p65 (p-p65) in placental tissues. It also inhibited excessive activation of the TLR4/NF-κB pathway, thereby reducing the release of inflammatory cytokines and alleviating inflammation-mediated placental injury [72]. In addition, AsIV also reduced inflammatory genes *IL-6* and *TNF-α* and suppressed NACHT, LRR, and PYD domain-containing protein 3 (NLRP3) inflammasome-related proteins in the pancreas of gestational diabetes mellitus (GDM) mice, leading to lower blood glucose and insulin levels [73].

The anti-inflammatory effects of AM can intervene in diabetes progression at an early stage. It also helps prevent cardiovascular and renal complications. When combined with conventional hypoglycemic drugs, it may enhance glycemic control through distinct mechanisms. Targeting inflammation is a key strategy for improving insulin sensitivity, protecting β-cell function, and preventing complications.

AsIV significantly reduces the levels of pro-inflammatory cytokines, including TNF-α, IL-6, and interleukin-8 (IL-8), in serum, thereby attenuating liver cell injury induced by inflammatory responses [74]. APSs inhibit the TLR4/NF-κB-NLRP3 pathway, restore intestinal barrier function, and thereby alleviate inflammatory responses. They also downregulate TLR4, p-NF-κB, and NLRP3, thereby suppressing the production and secretion of pro-inflammatory cytokines. Notably, these alterations in the gut microbiota exhibit significant correlations with lipid profiles as well as inflammatory mediators [75]. The progression of NAFLD is closely associated with excessive hepatic inflammation. Quercetin exerts anti-inflammatory effects through modulation of the adenosine monophosphate-activated protein kinase (AMPK)/MAPK/TNF-α signaling pathway. It enhances the phosphorylation of AMPK and markedly decreases p-p38, p-extracellular signal-regulated kinase (ERK), and phosphorylated c-JunN-terminal kinase (JNK). Furthermore, by inhibiting the MAPK pathway, quercetin significantly suppresses TNF-α mRNA expression in human hepatocellular carcinoma cell line (HepG2), thereby attenuating liver inflammation [76].

The alpha-7 nicotinic acetylcholine receptor (α7nAChR) serves as a critical component of the cholinergic anti-inflammatory pathway, and its activation directly suppresses the activation of the inhibitor of nuclear factor kappa-B kinase subunit beta (IKKβ)/NF-κB signaling cascade. AsIV downregulates both mRNA and protein levels of p-IKKβ and NF-κB via the α7nAChR. This downregulation reduces interleukin-1 beta (IL-1β) and TNF-α levels, thereby attenuating low-grade inflammation in the hypothalamus and adipose tissue. Consequently, AsIV inhibits excessive sympathetic nervous system activation, resulting in decreased blood pressure and heart rate [77].

Chronic low-grade inflammation and metabolic inflammation are often associated with metabolic changes directly related to CVD incidence, such as diabetes, hypertension, and obesity [78]. Zhang et al. [79] found that AsIV reduced lipid levels, decreased plaque area, and increased plaque stability in high-fat-diet-induced low-density lipoprotein receptor knockout mice. AsIV also reduced levels of inflammatory cytokines in serum, arterial, and hepatic tissues, as well as NF-κB p65 levels in the aortic root [79]. TFAs attenuate IL-1β and TNF-α through inhibition of the NF-κB signaling pathway, leading to a reduction in atherosclerotic lesion formation and enhanced plaque stability [80]. Sun et al. [81] reported that AsIV treatment significantly inhibited HG/heart failure (HF) and hypoxia-induced apoptosis of rat cardiomyoblast cell line (H9c2). It restored cardiac function and suppressed cardiac fibrosis and inflammation via the MAPK signaling pathway in diabetes [81]. You et al. found that AM extracts regulated adhesion molecule expression in endothelial cells through the NF-κB pathway, exhibiting anti-atherosclerotic effects [82]. Sun et al. [83] found that both atorvastatin (AV) and AsIV, as well as their co-administration, reduced p-p38/p38, inflammation-associated cytokines, with the most significant effects observed with the co-administration. These results suggest that AV and AsIV exert their anti-atherosclerotic effects by inhibiting the p38 signaling pathway, and the co-administration also exerted a potent anti-atherosclerotic effect via the NF-κB/peroxisome proliferator-activated receptor gamma (PPARγ) pathway [83]. AsIV reduces the mRNA expression of TNF-α and IL-6, thereby inhibiting the transcriptional activation of inflammatory pathways. Consistently, the protein levels of TNF-α and IL-6 in the cell culture supernatant are significantly decreased, which directly mitigates the detrimental effects of inflammatory factors on endothelial cells [84].

Fernandez et al. [85] studied TA-65 (a telomerase activator derived from AM in 40 patients with MetS). Participants were randomly assigned to receive either 16 mg/day TA-65 or a placebo, following a study design of two 12-week intervention periods separated by a 3-week washout phase. TA-65 administration significantly improves multiple cardiovascular risk markers: it increases high-density lipoprotein cholesterol (HDL-C), reduces body mass index (BMI) and waist circumference, optimizes the low-density lipoprotein (LDL)/HDL ratio, and decreases TNF-α levels [85]. Notably, the elevation of HDL-C is correlated with reduced inflammatory biomarkers. These findings suggest that TA-65 may confer cardiovascular benefits in MetS patients, which may be mediated by anti-inflammatory mechanisms.

#### 3.1.3. Regulating Intestinal Flora

In addition to its direct cellular effects, AM exerts influence on the pathogenesis of MetS through modulation of the gut microbiota.

APSs suppress the growth of potential pathogenic bacteria, such as *Shigella*, which can release LPS to trigger inflammatory responses. Additionally, APSs promote the proliferation of beneficial bacterial genera, including *Allobaculum* and *Lactobacillus*, thereby enhancing the integrity of the intestinal barrier [86]. Total astragalus saponins (TASs) increase microbial richness and enhance overall microbiota diversity. They reduce the Firmicutes/Bacteroidetes ratio by promoting the growth of beneficial bacteria such as *Bifidobacterium* while decreasing harmful bacteria such as *Lactobacillus*. Furthermore, TASs downregulate aberrantly activated Kyoto Encyclopedia of Genes and Genomes (KEGG) pathways associated with carbohydrate and lipid metabolism [87].

Xu et al. found that APSs modulated gut microbiota and restored microbial abundance in diabetic nephropathy (DN) rats [88]. Moreover, by regulating zona occludens-1 (ZO-1), occludin, and claudin-1, as well as increasing the relative abundance of beneficial bacteria, APSs are capable of enhancing intestinal barrier function. Beneficial bacteria such as *Lactobacillus* and *Faecalibacterium* produce short-chain fatty acids (SCFAs), which bind to G protein-coupled receptors (GPRs) and histone deacetylases (HDACs), thereby enhancing intestinal barrier function and reducing the translocation of harmful substances like LPS into the bloodstream [89,90]. The gut microbiota metabolizes APSs to produce high concentrations of SCFAs, which serve as key metabolic intermediates through which APSs exert their hypoglycemic effect. Moreover, SCFAs mediate glucagon-like peptide-1 (GLP-1) secretion via G protein-coupled receptor 43 (GPCR43) activation. GLP-1 is vital to the indirect hypoglycemic mechanism of APSs [91]. Gong et al. [92] observed that AsIV significantly reduced blood lipids and glucose levels in diabetic mice. The effect was mediated through regulation of the PI3K/Akt and AMPK/SIRT1 pathways, changes in gut microbiota composition, and increased butyrate levels [92].

He et al. [93] found that APSs effectively restored the increased abundance of proteobacteria and the decreased relative abundance of bacteroidetes and firmicutes caused by a high-fat diet. APSs also had a weight-loss effect on high-fat-fed mice, which was associated with the regulation of gut microbiota in obese mice [93]. Gut microbiota-derived SCFAs have been reported to offer a wide range of health benefits, including improvements in body composition and reduced body weight [94]. Zhao et al. found that APSs regulated the intestinal flora and increased intestinal SCFA content in rats with the syndrome of dampness stagnancy spleen deficiency (DSSD) [95]. AsIV can ameliorate obesity, dyslipidemia, and hepatic steatosis in mice. It does so by decreasing the expression and activity of intestinal bacterial bile salt hydrolase (BSH), as well as modulating intestinal bile acid composition, providing a viable pathway for the treatment of obesity and NAFLD [96].

Individual differences in drug efficacy are common. They may arise from variations in gut microbiota and host metabolic responses. However, the individual differences in the therapeutic effects of AM remain poorly understood. Their underlying mechanisms are also unclear. Wang et al. [97] integrated serum non-targeted metabolomics with 16S ribosomal RNA (16S rRNA) sequencing to characterize the metabolic profiles and gut microbiota of Institute of Cancer Research (ICR) mice. Among mice treated with AM (AR group), seven mice (AR-7 subgroup) showed significantly lower levels of aspartate transaminase (AST) and alanine transaminase (ALT) compared to the cisplatin (CDDP) group. This indicates that CDDP increases the abundance of *Deferribacteres*, which is a pro-inflammatory bacterial phylum. The results reveal significant disparities between the two AM-treated subgroups [97]. The disparities exist in biochemical markers, the extent of metabolite reversal, and microbiota modulation. This evidence supports a strong correlation. The correlation is between the host’s gut microbiota structure, metabolic traits, and AM’s therapeutic efficacy. The observed variability may stem from the influence of gut microbiota on drug metabolism. For example, gut microbiota can cause differences in fatty acid metabolism. However, the molecular mechanisms are still unknown. These mechanisms link gut microbiota to individual variability in AM response. This requires further investigation.

In short, AM has been shown to increase microbial diversity and restore microbial balance. Through the enhancement of intestinal barrier function, reduction in harmful substance translocation into the bloodstream, and elevation of the relative abundance of beneficial bacteria, AM effectively attenuates inflammation and oxidative stress. Given the high individual variability of gut microbiota, further studies are needed to clarify the interactions between specific AM components and key microbial taxa. Combining this with metabolomics may help reveal the links between microbial metabolites and host metabolic phenotypes in metabolic disease prevention and treatment.

#### 3.1.4. Regulating Lipid Metabolism

Dysregulation of lipid metabolism leads to excessive free fatty acids (FFAs) in the bloodstream, which in turn disrupts insulin signaling. FFA accumulation exacerbates oxidative stress, impairing insulin secretion and sensitivity. A prolonged high-fat environment accelerates pancreatic β-cell apoptosis. Lipid metabolism disorders also serve as catalysts for diabetic complications.

Cluster of Differentiation 36 (CD36) serves as a critical mediator of lipid uptake. AsIV attenuates lipid accumulation through the downregulation of CD36, leading to reduced expression of ferroptosis-related factors. In vitro studies showed that AsIV inhibited ferroptosis in cardiomyocytes. In vivo experiments demonstrated that AsIV improved myocardial contractility and reduced myocardial damage. Both types of studies provide evidence for the potential clinical application of AsIV in treating dilated cardiomyopathy (DCM) [98].

APSs inhibit the Wnt family member 1 (Wnt1) signaling pathway and upregulate lipid metabolism-related enzymes, including fatty acid synthase (FAS) and lipoprotein lipase (LPL), in liver tissue. This leads to enhanced lipid metabolism, reduced hepatic lipid deposition, and decreased serum total cholesterol (TC) and triglyceride (TG) levels [99]. Additionally, it lowered blood glucose levels in diabetic rats, an effect related to the inhibition of Wnt1 signaling [99]. Wang et al. [100] reported that APSs improved lipid metabolism disorders and sweetness receptor damage induced by a high-sugar and high-fat diet. This improvement occurred through the upregulation of sweetness receptor pathways, the promotion of intestinal hormone secretion (including GLP-1 and peptide YY), and a reduction in energy intake [100]. Guo et al. [101] observed that APS treatment significantly inhibited cell growth, as well as triglyceride and cholesterol levels in prostate cancer (PCa) cells. The treatment suppressed lipid metabolism via the miR-138-5p/SIRT1/sterol regulatory element-binding protein 1 (SREBP) pathways [101]. Wang et al. found that TFAs can significantly reduce TC, TG, and low-density lipoprotein cholesterol (LDL-C) levels, while increasing high-density lipoprotein (HDL-C) levels [102]. Moreover, TFAs also improved lipid and bile acid metabolism and lowered glucose levels in diabetic rats by regulating the farnesoid X receptor (FXR) and takeda G protein-coupled receptor 5 (TGR5) [102].

Huang et al. demonstrated that APSs reduced circulating leptin levels, potentially preventing weight gain by enhancing leptin sensitivity and modulating energy balance and appetite [103]. Li et al. reported that APSs improved lipid metabolism in obese mice through the microbial metabolite 2-hydroxybutyric acid, which effectively reduced body weight and obesity-related diseases in mice [104]. Wu et al. demonstrated that AsIV prevented obesity, reduced lipid content, and promoted fat oxidation in obese mice by interfering with the thermogenic network and alleviating leptin resistance [105]. Jin et al. revealed altered expression of fat mass and *obesity-associated gene* (*Fto*) and activating transcription factor 3 (Atf3) after treatment with AsIV [106].

Overall, AM and its active components can lower TG levels in the blood, reduce lipid deposition in liver tissue, and inhibit pancreatic cell apoptosis, thereby improving IR. The reduction in LDL-C and TG, along with the increase in HDL-C, can lower the risk of cardiovascular complications. Subsequent research ought to concentrate on monitoring the potential risks of hypoglycemia and hepatotoxicity when combined with lipid-lowering agents. Variations in lipid metabolism disorders among diabetic patients may influence the therapeutic outcomes.

AM achieves its therapeutic effects on MetS via a variety of pathways. These include exerting antioxidant effects, performing anti-inflammatory functions, regulating the composition of gut microbiota, and modulating lipid metabolism processes. These are closely interconnected and function synergistically to restore metabolic homeostasis. ROS can activate pathways including NF-κB, thereby promoting the secretion of pro-inflammatory cytokines. In turn, inflammatory mediators can further amplify ROS production, creating a self-perpetuating cycle. AsIV upregulates the Nrf2/HO-1 pathway, enhancing antioxidant enzyme activity and thereby reducing ROS levels. Additionally, AsIV suppresses the NF-κB pathway, resulting in decreased secretion of inflammatory cytokines. APSs also inhibit TLR4/NF-κB-mediated inflammatory responses. Gut dysbiosis is common in patients with MetS. This dysbiosis impairs the intestinal barrier. It also increases the translocation of LPS into systemic circulation. These changes thereby trigger systemic inflammation and oxidative stress. AM improves gut microbial composition by increasing the relative abundance of beneficial bacteria, which in turn produce higher levels of SCFAs. It also enhances the intestinal barrier by upregulating tight junction proteins such as ZO-1 and activates GPRs, thereby alleviating oxidative stress and inflammation. Dysregulation of lipid metabolism results in elevated levels of FFAs, which induce ROS generation via NADPH oxidase and activate inflammatory pathways such as TLR4/NF-κB. AM has been shown to alleviate redox imbalance and inflammation caused by excessive FFA accumulation. Furthermore, SCFAs activate AMPK, directly modulating lipid metabolism and reducing FFAs levels.

Although it is well established that AM exerts its effects on MetS through four major mechanisms—antioxidant activity, anti-inflammatory action, modulation of gut microbiota, and regulation of lipid metabolism—these mechanisms are not only interconnected but also function synergistically. However, the comprehensive regulatory network underlying its metabolic effects remains incompletely understood. Moreover, the interactions among these pathways have yet to be systematically elucidated. Based on current research, it is essential to identify core signaling pathways and biomarkers associated with the therapeutic effects of AM. To validate the synergistic interactions among multiple pathways, approaches such as gene knockout models and pathway-specific inhibitors could be employed. Further research should aim to refine and integrate the regulatory network involving antioxidant activity, anti-inflammatory responses, gut microbiota modulation, and lipid metabolism.

### 3.2. Therapeutic Effect on Diabetes

The pathophysiology of MetS is complex and involves multiple interrelated mechanisms. IR is one of the key factors. It contributes to pancreatic β-cell dysfunction and significantly increases the risk of type 2 diabetes. Epidemiological studies have shown that patients with MetS are five times more likely to develop type 2 diabetes compared to the general population [107].

#### 3.2.1. Improving Insulin Resistance

The characteristic of IR is the defect in insulin-mediated glucose metabolism control, especially in muscle, adipose tissue, and liver. AM treatment for IR is crucial for reversing this pathological state. AM regulates disorders of glucose metabolism through multiple targets and pathways, providing unique advantages for the treatment of MetS. Table 1 lists the mechanism of action of AM in treating IR.

Wu et al. found that APSs exerted insulin-sensitizing and hypoglycemic effects by reducing the elevated expression and the activity of protein tyrosine phosphatase 1B (PTP1B) in skeletal muscle of T2DM model rats [108]. Xu et al. later found that APSs promoted AMPK phosphorylation by suppressing PTP1B expression, thereby effectively alleviating IR [109]. Liu et al. showed that APSs increased adiponectin secretion and reduced IL-6 secretion in a dose-dependent manner in 3T3-L1 adipocytes derived from mouse embryonic fibroblasts [110].

**Table 1:** The mechanism of AM in improving IR.

NO	Model	Pathways	Effects *	Ref.
1	In vivo	/	↓ PTP1B	[108]
2	In vitro	/	↑ Adiponectin ↓ IL-6	[110]
3	In vivo	AMPK/PGC1α, IRS/AKT	↑ PGC-1α, p-AKT, p-AMPK, p-IRS-1 ↓ MCT4	[111]
4	In vitro	STAT5/IGF-1	↑ IGF-1R, p-AKT/AKT, IGF-1, p-STAT5/STAT5	[112]
5	In vivo	ROS-ERK-NF-κB	↓ myostatin, MDA, NF-κB	[113]
6	In vivo	ERS	↑ miR203a-3p ↓ GRP78	[114]
7	In vivo	SIRT1-PGC-1α/PPARα-FGF21	↑ FGF21, PPARα, SIRT1 ↓NF-κB	[115]
8	In vivo	/	↑ IRS-1, PI3K, PDK1, p-AKT ↓ p-GSK-3β	[87]
9	In vitro	C1q/CTRP3/PI3K/Akt	↑ p53, CTRP3 ↓ IL-6, TNF-α,	[70]
10	In vitro/ in vivo	JNK-AKT-GSK3β	↓ IL-6, TNF-α,	[116]
11	In vitro/ in vivo	/	↑ Adiponectin	[117]
12	In vitro	/	↑ p-IR, p-IRS-1 ↓ PTP1B	[118]
13	In vivo	/	↑ p-AMPK, HDL, ISI ↓ PTP1B, TG, TC, LDL, IS, IRI	[109]

* ↑ means increase; ↓ means decrease.

Zhang et al. demonstrated that AM improved cardiac IR and mitochondrial function by activating AMPK/peroxisome proliferator-activated receptor–gamma coactivator 1-alpha (PGC-1α) and insulin receptor substrate (IRS)/AKT pathways, thus exerting anti-heart failure effects [111]. APSs also increased the viability of HepG2 cells and alleviated IR by activating the signal transducer and activator of transcription 5/insulin-like growth factor 1 (STAT5/IGF-1) signaling pathway [112]. APS treatment improved hyperglycemia, hyperlipidemia, and IR in KKAy mice with non-insulin-dependent diabetes. It also downregulated myostatin expression and malondialdehyde production in skeletal muscle. This improvement was associated with the inhibition of the ROS-ERK-NF-κB signaling pathway [113]. Wei et al. suggested that APSs may alleviate IR in T2DM [114]. They may do so in three ways. First, they upregulate or maintain the expression of miR-203a-3p. Second, they decrease the mRNA and protein levels of GRP78. Third, they modulate the ERS pathway. In studies following eight weeks of catch-up growth in male Sprague Dawley rats, APSs suppressed abnormal glycolipid metabolism and IR. APSs activate the SIRT1-PGC-1α/PPARα-fibroblast growth factor 21 (FGF21) signaling pathway, leading to the upregulation of SIRT1, PPARα, and FGF21 expression. This activation promotes hepatic glucose and lipid metabolic homeostasis, thereby ameliorating IR [115].

Ma et al. reported that TAS from AM improved blood glucose levels, IR, and lipid profiles in T2DM rats [87]. In addition, TAS improved the morphology and structure of liver and colon tissues. In the liver, TAS upregulated the protein expression of insulin receptor substrate 1 (IRS-1), PI3K, 3-phosphoinositide-dependent protein kinase-1 (PDK1), and p-AKT. Meanwhile, TAS downregulated both the protein and mRNA expression of p-GSK-3β [87]. Zhang et al. [70] indicated that AsIV enhanced the expression of CTRP3, which subsequently activated the downstream PI3K/AKT signaling pathway. This activation promotes increased glucose uptake and upregulation of glucose transporter type 4 (GLUT-4), thereby ameliorating IR in adipocytes [70]. Ye et al. [116] demonstrated that AsIV reduced ROS, inhibited JNK phosphorylation, and restored the balance of downstream proteins. It upregulates PDK1 and AKT while downregulating GSK-3β, leading to the restoration of insulin signal transduction, enhanced glycogen synthesis, and improved glucose utilization [116]. Xu et al. found that astragaloside II and isoastragaloside I selectively enhanced adiponectin in primary adipocytes. This effect helped reduce hyperglycemia, glucose intolerance, and IR [117]. Similar to APSs, AsIV is also an effective and specific inhibitor of PTP1B. Zhou et al. demonstrated that AsIV suppressed PTP1B expression in insulin-resistant HepG2 cells and elevated the levels of phosphorylated insulin receptor (p-IR) and phosphorylated IRS-1 (p-IRS-1), thereby improving IR in these cells [118]. α-Astratide (aM1), a cystine-rich, proteolysis-resistant miniprotein isolated from AM by Dutta et al., can activate the PI3K/Akt signaling pathway [119]. It promoted GLUT4 translocation to the cell surface, enhancing glucose uptake. Additionally, it also altered gene expression to inhibit lipid synthesis and uptake, reducing lipid accumulation in myotubes and adipocytes. This prevented lipid-induced insulin receptor substrate1 and 2 (IRS1/2) degradation mediated by protein kinase C theta (PKCθ), thus restoring glucose uptake and overcoming IR [119].

APSs and astragalosides work synergistically to enhance insulin sensitivity. While each act through distinct mechanisms, their combined effect allows for coordinated regulation of multiple targets in the insulin signaling pathway, ultimately resulting in more robust improvements in insulin responsiveness. This cooperative interaction underscores the potential of AM as a therapeutic agent for IR-related conditions.

#### 3.2.2. Protection of Pancreatic β-Cell Function

Progressive dysfunction of pancreatic β-cells is a central pathological mechanism in type 2 diabetes. Preserving β-cell function can delay disease progression, reduce insulin dependence, improve glycemic control, and minimize complications related to oxidative stress and inflammation, such as vascular and neural damage. Table 2 lists the protective effect of AM on pancreatic islet β-cell.

Deng et al. [120] found that APSs improved proliferation and insulin secretion in mouse insulinoma 6 (MIN6) cells treated with high glucose and palmitic acid. This effect was mediated by upregulating miR-136-5p and miR-149-5p. These two miRNAs suppressed the expression of EF-hand domain family member D2 (EFHD2). This suppression thereby enhanced β-cell function under glucolipotoxicity conditions [120]. Zou et al. [121] demonstrated that APSs activated AMPK, promoting hepatic glycogen synthesis and glucose translocation in skeletal muscle. This reduced glucotoxicity and alleviated β-cell damage in a T2DM rat model [121]. Ren et al. [122] reported that APSs restored insulin secretion impaired by lipopolysaccharide-induced β-cell dysfunction. This effect was achieved through activation of the Akt/mammalian target of the rapamycin (mTOR)/GLUT2 signaling pathway [122]. Jiang et al. showed that AsIV protected against uric acid (UA)-induced pancreatic β-cell dysfunction via the activation of the PI3K/Akt pathway [123]. Lin et al. [124] treated streptozotocin (STZ)-induced insulinoma cells 1 (INS-1) cells with AsIV. The results demonstrated that the treatment reduced apoptosis, reversed the downregulation of B-cell lymphoma-2 (Bcl-2), and inhibited the upregulation of Bcl-2 antagonistX (Bax) and caspase-3. It also enhanced insulin secretion in damaged cells. These protective effects were associated with modulation of the SIRT1/p53 and Akt/GSK3β/Nrf2 signaling pathways [124].

**Table 2:** The protective effect of AM on pancreatic islet β-cell in vivo.

NO	Pathways	Effects *	Ref.
1	/	↑ miR-136-5p, miR-149-5p ↓ EFHD2	[120]
2	AMPK	↑ P-AMPKα, P-ACC, GLUT4	[121]
3	Akt/mTOR/GLUT2	↑ GLUT2, GCK, PDX-1, GSIS, p-Akt, p-mTOR	[122]
4	PI3K/AKT	↑ p-Akt, p-AKT/AKT ↓ caspase-3	[123]
5	SIRT1/p53 Akt/GSK3β/Nrf2	↑ Bcl-2 ↓ caspase-3, Bax	[124]

* ↑ means increase; ↓ means decrease.

#### 3.2.3. Increasing GLP-1 Levels

**Figure 6:**
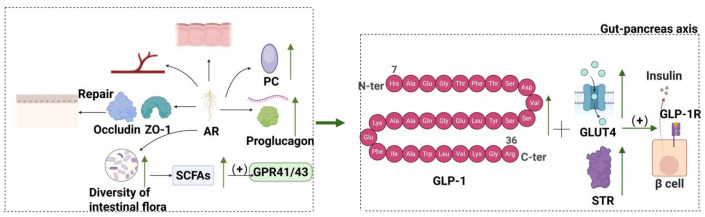
Regulation mechanisms of AM on GLP-1 (Green ↑ means increase).

GLP-1 is an intestinal insulinotropic hormone secreted by intestinal L-cells. Patients with type 2 diabetes often experience insufficient GLP-1 secretion or impaired action. AM and its active components can stimulate GLP-1 secretion and prolong its half-life, which has significant effects on blood glucose control and weight management. Figure 6 presents the regulation of GLP-1 levels by AM.

Huang et al. found that AsIV could significantly increase GLP-1 levels in the blood, intestinal tissues, and cells of rats [125]. It enhances the mRNA and protein expression levels of *proglucagon* and prohormone convertase (PC) in rat intestinal tissues and significantly improves the inhibitory effect of M-receptor blockers on GLP-1 cellular secretion. Several signaling pathways, such as AMPK, were involved in regulating intestinal GLP-1 secretion [125]. Furthermore, APSs significantly increased GLP-1 and sweet taste receptor (STR) signaling molecule expressions in the pancreas of T2DM rats, as well as GLP-1 receptor signaling molecule expressions [126]. APSs also alleviated T2DM rats by reversing the expression of glucose transporters and GLP-1/STR pathways in the intestine–pancreatic axis of T2DM rats [126]. Li et al. found that oral administration of TFAs reduced fasting blood glucose and food intake, repaired the blood–brain barrier, protected hippocampal synaptic function, improved hippocampal mitochondrial biosynthesis and energy metabolism, and enhanced hippocampal neuronal function in diabetic mice [127]. Meanwhile, the integrity of the intestinal barrier model was maintained. This maintenance of intestinal barrier integrity was mediated through the PGC-1α/AMPK pathway. These results suggest that astragalus flavonoids significantly improve brain damage by modulating the gut–microbiota–brain axis [127].

In general, AM and its active ingredients can increase the level of GLP-1 in blood and intestinal tissues and promote the secretion of GLP-1 by enhancing the expression of the *proglucagon* gene, PC protein, and tight junction proteins and activating G protein-coupled receptor 41/43 (GPR41/43). As a key signaling molecule of the gut–pancreatic axis, GLP-1 can bind to the receptors of pancreatic β-cells, upregulate the expression of STR signaling molecules, increase insulin secretion and the function of pancreatic islet β cells, and also regulate the expression of glucose transporters to improve glucose utilization (Figure 6).

AM and its constituent compounds exert therapeutic effects on diabetes-induced metabolic disorders through multiple mechanisms, including the improvement of IR, protection of β-cells, and regulation of intestinal microbiota. However, the majority of current mechanistic studies rely on animal models and in vitro cell systems, with limited clinical evidence available. Existing clinical trials are relatively scarce and generally involve small sample sizes. Individual active components of AM have been shown to ameliorate IR independently. However, the potential multi-target synergistic effects among flavonoids, saponins, and polysaccharides have not yet been validated. This validation needs to be carried out through orthogonal experimental designs. Future research should prioritize multi-center, randomized controlled trials (RCTs) involving larger cohorts and diverse subtypes of T2DM patients. Additionally, network pharmacology integrated with animal studies should be employed to design various combinations of flavonoids, saponins, and polysaccharides, aiming to elucidate the molecular mechanisms underlying their synergistic multi-target regulation of core signaling pathways.

### 3.3. Anti-Nonalcoholic Fatty Liver Disease (NAFLD)

Metabolic dysfunction-associated fatty liver disease (MAFLD), formerly known as NAFLD, is a condition characterized by excessive fat accumulation in the liver unrelated to alcohol consumption [128,129]. Diabetes and NAFLD frequently coexist in MetS, sharing common pathological mechanisms such as IR and dysregulated lipid metabolism. Given that AM has demonstrated beneficial effects on glucose metabolism, its potential therapeutic role in NAFLD warrants further investigation. NAFLD is a strong predictor for the future development of MetS, with significant clinical implications for the diagnosis, prevention, and treatment of the syndrome [130]. Table 3 lists the mechanism of AM in treating metabolic disorders of NAFLD.

Zhao et al. [131] utilized network pharmacology techniques to predict the targets, pathways, and mechanisms of AM in treating MAFLD. They discovered that the multiple active ingredients of AM could synergistically act through various targets and pathways, playing a therapeutic role in MAFLD through anti-inflammatory effects, regulation of lipid metabolism, and modulation of oxidative stress [131]. Zhou et al. demonstrated for the first time that AsIV treated hepatic steatosis by alleviating FFA-induced ERS and lipid accumulation via AMPK activation in hepatocytes [132]. Wei et al. found that AsIV improved hepatic lipid deposition in NAFLD mice by directly activating AMPK [133]. Yang et al. [134] constructed a NAFLD mouse model with a high-fat, high-sugar diet and administered various concentrations of AsIV. The results showed that AsIV could reduce liver lipid accumulation, with the most significant effect observed at a concentration of 60 mg/kg [134].

**Table 3:** The mechanism of AM in treating metabolic disorders of NAFLD.

NO	Model	Pathways	Effects *	Ref.
1	In vitro	/	↑ AMPK, ACC, SREBP-1c ↓ acc1, fas, scd1, GRP78, CHOP	[132]
2	In vivo	TLR4/NF-κB	↓ AST, ALT, TG, TNF-α, IL-6, IL-8, TLR4, MyD88, NF-κB	[74]
3	In vitro/ in vivo	/	↑ GSH-Px, Bcl-2, Bax ↓ ROS, MDA, 5-LO, LTB4	[62]
4	In vivo	SCFA-GPR	↑ZO-1, occludin ↓ TLR4, NF-κB, NLRP3, GPR	[75]
5	In vitro/ in vivo	/	↑ THDCA, CYP7B1 ↓ CYP7A1, CYP8B1	[135]
6	In vivo	FXR	↓ BG, TG, HBA	[136]
7	In vitro	AMPK/MAPK/TNF-α, AMPK/ACC/CPT1α	↑ AMPK, ACC, CPT1α ↓ p-MAPK, TNF-α	[76]

* ↑ means increase; ↓ means decrease.

Liu et al. demonstrated that AsIV significantly alleviated hepatic steatosis, reduced lipid droplet accumulation in hepatocytes, and improved liver injury in NAFLD rats through inhibition of the TLR4/myeloid differentiation primary response 88 (MyD88)/NF-κB signaling pathway [74]. Zhong et al. [75] revealed that APSs ameliorated hepatic steatosis via the AMPK-PPAR-α signaling pathway. They enhanced the protein and mRNA expression levels of p-AMPK and PPAR-α, while suppressing SREBP-1 expression, leading to reduced hepatic TC and TG content as well as decreased lipid droplet accumulation [75]. Zheng et al. showed that APSs regulated serum and liver bile acid profiles in high-fat-diet-fed mice, increasing serum taurohyodeoxycholic acid (THDCA), reducing hepatic lipid accumulation, improving glucose homeostasis, and decreasing triglyceride levels in alpha mouse liver 12 (AML-12) cell line treated with palmitic acid/oleic acid [135]. Gu et al. found that cycloastragenol (CAG), a natural molecule from AM, effectively treated NAFLD by activating the nuclear receptor FXR signaling in vivo and in vitro [136]. Fu et al. [76] confirmed that quercetin, the active component of *A. mongholicus* Bunge (AMB), promoted fatty acid β-oxidation and attenuates hepatic lipid deposition via the AMPK/acetyl-CoA carboxylase/carnitine palmitoyltransferase 1α (ACC/CPT1α) pathway. Its mechanism of action includes upregulating p-AMPK and inducing phosphorylation of ACC. Inhibition of ACC activity results in decreased production of malonyl-CoA, a critical intermediate in fatty acid synthesis, thereby alleviating the suppression of CPT1α, a key regulatory enzyme in fatty acid β-oxidation [76].

**Figure 7:**
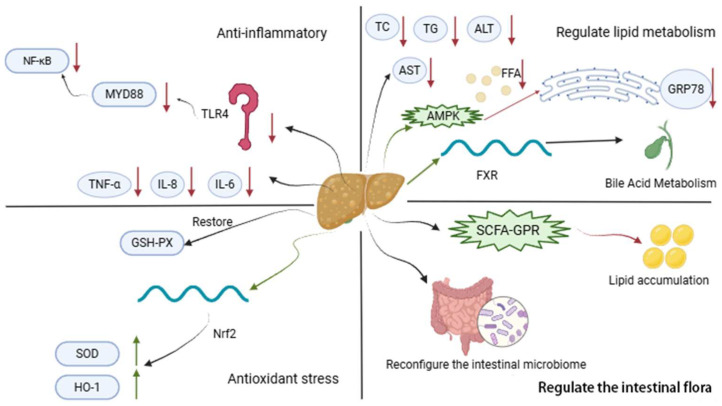
Mechanism of AM multi-target intervention in NAFLD (Green ↑ means increase; red ↓ means decrease).

To sum up, the main aspects of AM in the treatment of NAFLD are related to anti-inflammation, regulation of lipid metabolism, anti-oxidative stress, and regulation of intestinal flora (Figure 7).

Generally, AM can improve lipid metabolism through multiple pathways, such as activating AMPK to inhibit lipid synthesis and reducing hepatic lipid accumulation, or modulating bile acid homeostasis and affecting FXR signaling. AM also enhances antioxidant capacity by upregulating enzymes like GSH-Px to clear lipid peroxides such as MDA, protecting hepatocytes from oxidative damage. Furthermore, AM reduces the release of inflammatory cytokines, alleviating liver inflammation. Its multi-target effects align with the complex pathogenesis of NAFLD that often coexists with T2DM. AM’s glucose-lowering and lipid-lowering effects contribute to the comprehensive management of MetS.

Subsequent research ought to concentrate on elucidating the specific regulatory mechanisms of AM and its components on the gut microbiota–intestinal–liver axis. The potential variation in the therapeutic effects of AM across the progression of NAFLD—from simple steatosis to non-alcoholic steatohepatitis (NASH) and eventually liver fibrosis—remains poorly understood, primarily due to the lack of stage-specific investigations. Obeticholic acid, a commonly prescribed agent for NAFLD in clinical settings, is associated with adverse effects such as diarrhea. Whether co-administration of AM with obeticholic acid can enhance therapeutic efficacy while mitigating side effects has yet to be validated in animal models. Furthermore, no studies have explored the relationship between AM-induced improvements and established serum biomarkers of NAFLD, such as fibroblast growth factor 21 (FGF21) and cytokeratin-18 (CK18), which limits the ability to quantitatively assess its intervention effects during disease progression. Future research should aim to identify the specific bile acid metabolites resulting from key microbial alterations following AM treatment. In experimental models representing different stages of NAFLD, the impact of AM components on hepatic lipid accumulation and fibrosis markers should be systematically compared to determine stage-specific active constituents. Additionally, combining therapy groups involving both obeticholic acid, AM should be included in NAFLD models to assess markers of liver injury and adverse effects, thereby evaluating potential synergistic detoxification effects. Finally, clinical studies should be expanded to measure serum levels of FGF21 and CK18 after AM intervention, providing much-needed quantitative biomarkers for evaluating clinical efficacy.

### 3.4. Therapeutic Effect on Obesity

Obesity is one of the core features of MetS. It is not only an important inducement of NAFLD but also closely associated with IR. While AM improves hepatic lipid accumulation, its intervention on obesity essentially blocks the upstream pathological mechanisms such as IR through multi-target synergy. Over 1.9 billion adults worldwide were overweight or obese. Childhood obesity has been proven to increase the risk of developing type 2 diabetes, hypertension, dyslipidemia, atherosclerosis, and related CVD in adulthood [137].

Ma et al. demonstrated that APSs could inhibit lipolysis and the browning of white adipose tissue and reduce the thermogenesis of brown adipose tissue, thus inhibiting obesity [138]. Zhang et al. [139] found that APSs affected the expression of long non-coding RNAs (lncRNAs) during brown adiposity in mouse mesenchymal stem cells. By regulating differentially expressed lncRNAs (DElncRNAs), APS influences the relative expression of proteins in signaling pathways, including glucagon and cyclic adenosine monophosphate (cAMP), to promote brown adipose differentiation [139]. Cao et al. suggested that APSs enhanced the differentiation of C3H/10T1/2 mouse embryo fibroblast cell line (C3H10T1/2) into brown adipocytes and induced the expression of key brown adipogenic transcriptional factors through regulating cut-like homeobox 1 (Cux1) via miR-1258-5p [140]. Nie et al. [141] demonstrated that formononetin activated PPARγ, which subsequently formed a heterodimer with the retinoid X receptor (RXR). This heterodimer directly binds to the promoter region of the *uncoupling protein 1* (*UCP1*) gene. This binding promotes the upregulation of UCP1 expression. It also increases the mRNA levels of other thermogenesis-associated genes. These genes include *PR domain-containing protein 16* (*PRDM16*), *PGC-1α*, *iodothyronine deiodinase 2* (*Dio2*), and *cell death-inducing DFFA-like effectora* (*Cidea*). This process ultimately enhances the synergistic activation of the thermogenesis pathway [141]. Long et al. found that AM ameliorated high-fat-diet-induced disturbances in triglyceride metabolism and attenuated adipose tissue macrophage infiltration in obese male rats by activating the mechanistic target of rapamycin complex 1 (mTORC1)-PPARγ signaling pathway [142]. Kim et al. [143] showed that CAG significantly reduced body weight gain in experimental models. Notably, it did not affect brown fat weight. In visceral white adipose tissue (vWAT), CAG regulated the expression of three key molecules. These molecules are PPARγ, C/EBPα, and recombinant glioma-associated oncogene homolog 1 (Gli1). This regulatory effect of CAG was mediated through the hedgehog signaling pathway [143].

APSs regulate fat browning by upregulating the expression of uncoupling protein 1 (UCP1), increasing energy expenditure. AsIV improves IR, leptin resistance, and promotes lipid oxidation. By working synergistically, these components improve multiple metabolic abnormalities and block the progression of obesity to MetS. Additionally, AM can regulate gut microbiota imbalances related to obesity, enhance SCFA production, and indirectly improve metabolic disorders.

AM alleviates obesity by modulating the composition of the gut microbiota and enhancing the production of SCFAs. However, this relationship has been primarily described in qualitative terms and lacks quantitative investigations linking SCFAs levels to energy metabolism. Notably, significant gender differences exist in the pathophysiological mechanisms of obesity; for example, estrogen levels play a crucial role in female obesity [144]. Future studies should incorporate precise quantification of SCFA concentrations to determine the threshold levels required for the activation of key metabolic regulators such as AMPK and UCP1. Furthermore, gender-stratified experimental designs should be implemented to evaluate the effects of AM on body fat distribution and estrogen signaling, with a focus on elucidating the potential interactions between AM-derived bioactive components and estrogen receptors.

### 3.5. Therapeutic Effect on Hypertension

Obesity is frequently comorbid with hypertension, and their coexistence significantly exacerbates the burden on the cardiovascular system. AM also exerts a regulatory effect on MetS through multi-target mechanisms, with a notable intervention on hypertension as a key component. Hypertension is a major global public health issue and a leading reversible cause of cardiovascular disease [145]. Controlling blood pressure is a core aspect of MetS management, which is of great significance for reducing the risk of cardiovascular events, improving metabolic disorders, and delaying multi-organ damage.

Li et al. found that long-term administration of small doses of AM reduced arterial stiffness and urinary microalbumin in patients with hypertension and MetS [146]. Liu et al. [147] suggested that AM might lower blood pressure by dilating blood vessels and reducing cardiac output. It also significantly reduced total cholesterol and triglyceride levels in the serum of hyperlipidemic mice, thereby lowering blood lipids [147]. In a mouse model of hypertension, APSs significantly reduced systolic blood pressure. They directly modulated the transforming growth factor-beta1-integrin-linked kinase (TGF-β1-ILK) signaling pathway, leading to decreased protein expression of both TGF-β1 and ILK. This downregulation inhibits fibroblast activation and the transformation into myofibroblasts. By protecting renal structure and function, APSs restored serum creatinine levels to the normal range and decreased the levels of urea nitrogen and uric acid. Overall, APS exerts multi-dimensional protective effects against hypertension-induced renal injury, encompassing blood pressure reduction, fibrosis inhibition, and improvement of renal function [148]. AsIV prevented weight gain and fat accumulation, ameliorated metabolic disorders in high-fat diet rats, and improved hypothalamic leptin resistance. It did so by increasing the expression of the α7nAChR in central and peripheral tissues and inhibiting the IKKβ/NF-κB signaling pathway. This reduces sympathetic-mediated obesity-associated hypertension [149]. AsIV upregulates the expression of hypothalamic leptin receptor (LepRb) mRNA and promotes signal transducer and activator of transcription 3 (STAT3) phosphorylation. Simultaneously, it reduces the mRNA levels of suppressors of cytokine signaling 3 (SOCS3) and PTP1B, as well as the expression of p-PI3K, thereby restoring leptin’s regulatory capacity over energy metabolism. Through the improvement of leptin resistance, AsIV indirectly suppresses sympathetic nervous system activation, attenuates the excessive sympathetic drive on the cardiovascular system, and ultimately reduces blood pressure and heart rate [77]. Li et al. [150] performed a randomized controlled trial involving 226 patients with essential hypertension (EH) complicated by MetS, who were divided into three groups. The control group (*n* = 76) received conventional medical treatment only; the high-dose AM group (*n* = 74) received conventional treatment plus 10 g/day AM extract capsules; and the low-dose AM group (*n* = 76) received conventional treatment plus 5 g/day AM extract capsules. The study aimed to evaluate the early cardiorenal protective effects of different AM dosages. After 12 months of treatment, urinary microalbuminuria (MAU) decreased in all groups, with the most significant reduction in the high-dose AM group (significantly greater than that in the control and low-dose AM groups). Additionally, the high-dose AM group showed a significant decrease in end-systolic volume (ESV) post-treatment, while both AM groups had a significant increase in mitral valve peak velocity [150]. These results indicate that AM combined with conventional therapy exerts early cardiorenal protection in patients with EH and MetS, and the high dose (10 g/day) has a more prominent protective effect than the low dose.

In conclusion, AM exerts regulatory effects on hypertension and related metabolic disorders through various mechanisms, including anti-inflammatory action, metabolic improvement, lipid lowering, and kidney protection. These findings provide scientific evidence for the use of AM in hypertension treatment and suggest that it may become an effective adjunctive therapeutic approach.

Current research remains limited to qualitative associations. For example, AM shows to upregulate α7nAChR expression and concurrently reduce blood pressure. However, the lack of a clear quantitative relationship between these two factors hinders the determination of effective clinical dosages. Given that long-term pharmacotherapy is essential for managing hypertension combined with MetS, existing clinical studies on AM suffer from several limitations, including small sample sizes, short observation periods, and restricted outcome measures. Future research should prioritize multi-dose animal experiments to establish the quantitative correlations among AM component dosages, α7nAChR activation intensity, and the magnitude of blood pressure reduction. Furthermore, large-scale randomized controlled trials are needed to rigorously evaluate the vascular protective effects associated with long-term, low-dose administration of AM.

### 3.6. Therapeutic Effect on Cardiovascular Disease

Hypertension represents a primary risk factor for CVD. The dynamic progression of metabolic MetS shows to significantly influence the risks of CVD, stroke, and mortality, with prolonged MetS markedly amplifying these outcomes [151]. Although AM exhibits blood pressure-regulating properties, its protective effects and underlying mechanisms in CVD require further elucidation to establish a clear therapeutic foundation.

#### 3.6.1. Improving Endothelial Function

TFAs improve atherosclerosis by reducing aortic lesion area and enhancing plaque stability. This effect is primarily mediated through the downregulation of miR-33 expression, which alleviates the suppression of ATP-binding cassette transporter A1 and G1 (ABCA1/G1) and promotes cholesterol efflux. Additionally, TFAs inhibit scavenger receptors CD36 and scavenger receptor class A (SRA), thereby reducing cholesterol uptake [80]. Astragali radix extract suppresses the NF-κB signaling pathway by reducing phosphorylated inhibitor of kappa B (p-IκB), thereby preventing NF-κB nuclear translocation. This leads to decreased expression of vascular cell adhesion molecule-1 (VCAM-1) and intercellular adhesion molecule-1 (ICAM-1) in endothelial cells, reduced adhesion of monocytes to endothelial cells, and ultimately inhibiting the initiation and progression of atherosclerosis [82]. AM exhibits to improve impaired endothelium-dependent vasodilatory function and protect the cardiovascular system in high-fat-fed obese rats. This effect is linked to alleviating IR and directly promoting increased NO production in endothelial cells [152]. Meng et al. [153] found that AsIV exerted three key effects. First, it prevented or reversed the uncoupling of eNOS. Second, it increased the levels of eNOS and NO. Third, it enhanced eNOS enzyme activity. These effects thereby activated the antioxidant system. They also improved oxidative stress-mediated endothelial dysfunction in cardiovascular disease [153]. Zhu et al. [84] demonstrated that AsIV increased mRNA expression of Nrf2 and HO-1, while decreasing mRNA expression of TNF-α and IL-6 in ox-LDL-treated human umbilical vein endothelial cells. AsIV prevented ox-LDL-induced endothelial cell injury by reducing apoptosis, oxidative stress, and inflammatory responses [84]. AsIV also facilitated the migratory and angiogenic functions of human endothelial progenitor cells (EPCs) in vitro. Additionally, AsIV inhibited thrombosis and reduced leukocyte infiltration into the thrombus and the production of pro-inflammatory cytokines in rats through inactivation of the PI3K/AKT signaling pathway [154]. Moreover, APSs ameliorated hyperhomocysteinemia (HHcy)-induced endothelium-dependent vasodilatory dysfunction through blood pressure regulation, matrix metalloproteinase (MMP) activity, and antioxidant effects [155].

Li et al. [156] evaluated the effect of different dosages of AM on left ventricular diastolic dysfunction (LVDD) in postmenopausal women with hypertension and MetS. The study showed that conventional drug therapy combined with AM significantly improved left ventricular diastolic function. However, 5 g/day AM did not produce a statistically significant improvement compared with higher AM dosages. These results confirm that the therapeutic effect of AM on diastolic function is dose-dependent [156].

#### 3.6.2. Autophagy Regulation

Basal level autophagy is essential for removing damaged cellular components and maintaining lipid homeostasis. Autophagy and lipophagy have been considered novel therapeutic targets for cardiometabolic syndrome and atherosclerosis due to their regulatory effects on lipid metabolism [157]. Table 4 lists the action mechanism of AM in treating CVD. Wang et al. [158] suggested that AsIV could protect against LPS-induced cardiac dysfunction. It exerts protective effects by inhibiting calcium-mediated apoptosis and autophagy. This inhibition is achieved through targeting miRNA-1. This finding highlights a new mechanism for the therapeutic effect of AsIV on cardiac dysfunction [158]. Zhang et al. showed that AsIV decreased the expression of NLRP3 and IL-1β, activated autophagy, and improved cardiac function and cardiomyocyte morphology, limiting hypertrophy [159]. AsIV also alleviated hypoxia/reoxygenation (H/R)-induced apoptosis and autophagy in rat cardiomyoblast (H9c2) cell line and stimulated the overexpression of recombinant GATA binding protein (GATA)-4 [160].

**Table 4:** The mechanism of AM in treating CVD.

NO	Pharmacology	Diseases	Model	Pathways	Effects *	Ref.
1	Anti-atherosclerotic	AS	In vivo	MAPK/NF-κB	↓ NF-κB, p65, JNK, ERK1/2, p38, iNOS, VCAM-1, IL-6	[79]
2			In vitro/ in vivo	miR-33, NFκB	↑ ABCA1/G1 ↓ CD36, SRA, miR-33, FκB	[80]
3			In vitro	NF-κB	↓ VCAM-1, ICAM-1, p-iκB, NF-κB	[82]
4			In vivo	NF-κB/PPARγ	↑ PPAR-γ ↓ oxLDL, TNF-α, IL-6, IL-18, NF-κB, CD36, MMP-9, ICAM-1, VCAM-1, P-p38	[83]
5			In vivo	/	↑ GPR78, CHOP, LC3-II;, beclin-1, ATG5 ↓ ER	[66]
6	Anti-myocardial infarction	MI	In vitro/ in vivo	MAPK, EKR, JNK	↑ EKR ↓ JNK, p38	[81]
7	Vascular protection	HHcy	In vivo	/	↓ MMP-2, MMP-9	[155]
8	Anti-heart failure	HF	In vivo	/	↓ miR-1	[158]
9	Activate autophagy	MH	In vivo	/	↑ LC3-II ↓ NLRP3, IL-1β, p62	[159]
10	Protect cardiomyocytes	/	In vitro	/	↑ GATA-4, Bcl-2, p62 ↓ PARP, Caspase-3, Beclin-1, LC3-II	[160]

* ↑ means increase; ↓ means decrease.

AsIV can reduce the area of arterial plaques and enhance their stability by inhibiting inflammatory pathways, improving endothelial function, and reducing oxidative stress-induced endothelial damage. It can also promote autophagy to decrease macrophage apoptosis and lipid deposition in plaques. Furthermore, TFAs and AsIV act synergistically in inhibiting inflammation, while APSs improve endothelial dysfunction and enhance plaque stability through mechanisms distinct from those of AsIV. The multi-target effects of AsIV and its active components provide a new strategy for the treatment of CVD, particularly in inflammation and plaque stability.

Existing studies have demonstrated that AsIV can reduce macrophage apoptosis and enhance collagen content in atherosclerotic plaques. However, its long-term efficacy in preventing plaque rupture and reducing the risk of acute cardiovascular and cerebrovascular events remains to be validated. AM exerts a bidirectional regulatory effect on autophagy; however, the threshold at which AM switches between activation and inhibition of autophagy has not yet been clearly defined. In the treatment of CVD, the concurrent use of AM and statins raises questions regarding potential metabolic competition in the liver and other possible side effects, which remain insufficiently investigated. Moreover, most current animal studies predominantly employ male models, leaving the potential influence of AM on therapeutic outcomes through estrogen receptor modulation unexplored.

Future research should include long-term follow-up studies to assess plaque stability under continuous AM intervention, with dynamic monitoring of plaque area and rupture rates. Additionally, pharmacokinetic–pharmacodynamic (PK-PD) interactions between AM and statin therapy should be systematically evaluated. Gender should be incorporated as a key variable in future investigations. Specifically, animal experiments should utilize MetS models with equal representation of male and female subjects to compare the cardiovascular effects of AM and analyze estrogen receptor-mediated differential signaling pathways.

### 3.7. Therapeutic Effect on Other Diseases

In addition to the core components of MetS, this condition is frequently associated with other metabolic disorders, including hyperuricemia and polycystic ovary syndrome (PCOS). AM has demonstrated potential in modulating these comorbidities, thereby contributing to a more comprehensive therapeutic strategy for MetS.

Deng et al. [161] found that AM modulated the composition of the intestinal microbiota and associated metabolic pathways, resulting in decreased levels of serum UA, xanthine oxidase activity, creatinine, blood urea nitrogen (BUN), and hepatic xanthine oxidase (XOD), as well as reduced ALT/AST ratios. Moreover, it exerts renoprotective effects by ameliorating renal tubular dilation [161]. He et al. [162] suggested that AsIV could prevent kidney injury in rats, which is induced by iatrogenic hyperinsulinemia. AsIV exerts this renal protective effect by inhibiting oxidative stress. It also reduces the overproduction of IL-1β and TNF-α, while downregulating the activation of ERK1/2. Additionally, it upregulates the protein expression of transient receptor potential cation channel l6 (TRPC6) [162]. Wang et al. [163] found that *Bacillus subtilis*-fermented *Astragalus membranaceus* (BFA) could alter the gut microbiota composition in hyperuricemic mice. Specifically, BFA increased the abundance of butyrate-producing bacteria. These include *Butyricimonas synergistica*, *Odoribacter splanchnicus*, and *Collinsella tanakaei*. It also elevated the abundance of probiotics, such as *Lactobacillus intestinalis* and *Bacillus mycoides* [163]. Zhang et al. indicated that astragali radix could promote UA excretion by regulating UA transporters via the PI3K/Akt signaling pathway, providing experimental and clinical support for its use in treating hyperuricemia [164]. AM alleviated hyperuricemia (HUA) through mechanisms such as bile acid metabolism, hormone synthesis, and fatty acid absorption [165]. Wang et al. found that *paecilomyces cicadae*-fermented radix astragali treated HUA by lowering serum uric acid, triglycerides, glucose, and creatinine levels, as well as reducing renal inflammation and lipid accumulation in the liver [166].

Nejati et al. demonstrated that *Astragalus hamosus* extract-treated groups showed decreased levels of insulin and testosterone compared to the PCOS group. Moreover, *Astragalus hamosus* extract improved PCOS by influencing IRS1 expression [167]. Li et al. showed that APSs improved PCOS in mice by correcting serum metabolic disorders and enhancing microbiome diversity, providing insights into its potential therapeutic effects for PCOS [168]. Wen et al. found that AsIV could promote autophagy, inhibit proliferation, and promote apoptosis of ovarian granulosa cells by activating the PPARγ signaling pathway, ultimately improving ovarian function in rats with PCOS [169]. Table 5 lists the therapeutic mechanisms of AM on other diseases.

To sum up, AM and its active ingredients exhibit extensive therapeutic potential in various metabolic diseases. In hyperuricemia, fermented products of AM restore microbiota balance, indirectly modulate bile acid and lipid metabolism pathways, and lower serum uric acid levels. AsIV inhibits uric acid reabsorption transporters, thereby promoting uric acid excretion. Additionally, AsIV protects the kidneys from high insulin-induced damage by reducing type IV collagen and laminin deposition and delaying kidney fibrosis progression. In PCOS, AM extract alleviates hormonal imbalances, while APSs improve PCOS symptoms by regulating glucose and lipid metabolism and gut microbiota. AsIV aids in restoring ovarian function by promoting autophagy and apoptosis in ovarian granulosa cells. The multi-target, multi-pathway actions of AM and its active components show broad potential in treating metabolic diseases. However, some key issues still need further investigation, such as verifying the direct link between microbiota regulation and uric acid metabolism, understanding the effects of different fermentation processes on efficacy, exploring the connection between the PPARγ pathway and ovarian hormone secretion, and uncovering the interactions within the microbiota–metabolism–endocrine axis.

**Table 5:** The therapeutic mechanism of AM on other diseases.

NO	Pharmacology	Diseases	Model	Pathways	Effects *	Ref.
1	Anti-hyperuricemia	HU	In vivo	/	↓ UA, XOD, CRE, ALT/AST, BUN	[161]
2			In vitro/ in vivo	PI3K/Akt	↑ ABCG2 ↓ URAT1, GLUT9	[164]
3	Anti-hyperinsulinemia	HI	In vivo	/	↑ TRPC6, GPx, SOD, NOX4 ↓ ERK1/2, MDA, IL-1β, TNF-α, IV Collagen, Laminin	[162]
4	Anti-PCOS	PCOS	In vivo	/	↑ CL, IRS1; ↓CF, INS, T	[167]

* ↑ means increase; ↓ means decrease.

Current studies predominantly rely on animal models. However, there remains a critical gap in large-sample RCTs to substantiate the therapeutic efficacy of AM. Small-scale or observational studies are insufficient to establish reliable dosing recommendations or to assess the long-term safety profile of AM in clinical settings. Furthermore, existing PCOS animal models fail to fully recapitulate the core pathophysiological features of human PCOS, particularly the integrated manifestations of hyperandrogenism, ovulatory dysfunction, and IR. These limitations significantly constrain the clinical translational value of the research findings. Additionally, the mechanistic understanding of AM’s therapeutic actions remains incomplete, with key molecular pathways and their regulatory nodes yet to be fully elucidated. To address these gaps, well-designed, multicenter clinical RCTs are warranted to determine the optimal therapeutic dosage and safety profile of AM in human patients. Complementary in vitro studies using cell models should be employed to validate the regulatory mechanisms of critical nodes within the proposed molecular pathways.

**Figure 8:**
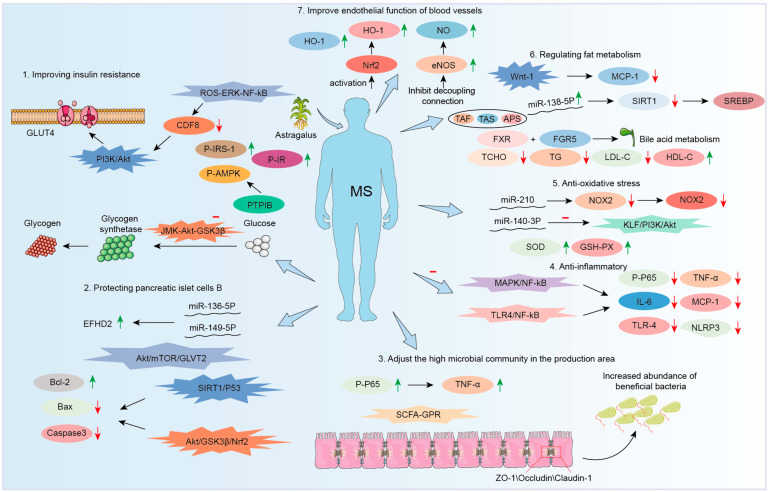
Mechanisms of AM in treating MetS (Green ↑ means increase; red ↓ means decrease/suppress).

In summary, the main aspects of AM treatment for MetS include improving IR, protecting pancreatic beta cells, regulating the intestinal flora, anti-inflammation, anti-oxidative stress, regulating lipid metabolism, and improving vascular endothelial function (Figure 8).

## 4. Study on the Stems and Leaves of AM

The preceding discussion has comprehensively outlined the therapeutic potential of AM in managing MetS. However, during the industrialization process, its above-ground parts—primarily stems and leaves—are frequently discarded or merely used for composting. These components contain bioactive compounds similar to those found in the roots, yet their medicinal value remains largely untapped and warrants further exploration.

Cui et al. [170] optimized the extraction and isolation of flavonoids from the stems and leaves of AM. The optimal extraction process was determined to be an extraction time of 35 min, an ethanol concentration of 75%, a liquid-to-solid ratio of 40 mL/g, and an extraction temperature of 58 °C. Ultrasonic-assisted extraction (UAE) combined with the response surface methodology (RSM) can efficiently extract flavonoids. The highly active components are mainly isoquercitrin and astragaloside. These components possess significant antioxidant and antifungal activities and can serve as natural food antioxidants and preservatives [170]. The flavonoid components from the stems and leaves of AM exhibit inhibitory effects on *Bacillus cereus*. The main active ingredient is isoliquiritigenin. It disrupts the bacterial structure, inhibits biofilm formation, interferes with protein synthesis, and downregulates virulence genes. This provides a foundation for the resource utilization of the stems and leaves of AM [171]. Astraside D is a new flavonol glycoside extracted and isolated from AM. It exerts cytotoxic effects on rat pheochromocytoma (PC12) cell line at concentrations ranging from 5 to 100 μM. Additionally, it significantly inhibits NO production in LPS-stimulated immortalized mouse microglial (BV2) cell line microglial cells, indicating potential anti-inflammatory activity [37]. Cyclastraine A exhibited significant biological activities: it remarkably reduced the levels of pro-inflammatory cytokines (IL-6, IL-1β, and TNF-α) in cell supernatants, decreased the percentage of apoptotic cells, and downregulated the expression of apoptosis-related proteins (Caspase-3, Bax, and cytochrome C) [40]. Samuel et al. [172] compared the bioactive components and biological activities of the dried leaves, leaf tea, and dried roots of AM, aiming to evaluate the resource utilization potential of its leaves. The contents of total flavonoids, total polyphenols, proteins, and amino acids in the dried leaves and leaf tea were significantly higher than those in the dried roots. Furthermore, the dried leaves and leaf tea exhibited remarkably superior antioxidant and antibacterial activities compared with the dried roots [172]. Therefore, processing AM leaves into leaf tea can serve as an effective strategy for the resource utilization of this plant part. Nevertheless, this study lacks a comparative analysis of saponin components. Additionally, the AM samples are sourced from only a single geographical origin. Notably, the biological activities and components of AM may vary with geographical origin. It is also important to highlight that this study is restricted to in vitro investigations; consequently, the safety of long-term consumption of AM leaves or leaf tea remains unascertained. Growing evidence shows that these plant parts can act as feed additives, effectively improving poultry growth performance and enhancing their immune function. Furthermore, their favorable safety profile supports their application as raw food materials or dietary supplements.

The exploitation and utilization of the stems and leaves of AM are still at a preliminary stage. Currently, there is a lack of in vivo pharmacodynamic studies. These studies use MetS animal models to evaluate the efficacy of AM stems and leaves. Whether these aerial parts have equivalent effects to AM roots is uncertain. Specifically, their effects on improving IR and modulating lipid profiles need to be confirmed. Additionally, the pharmacological potential of newly identified bioactive compounds requires further investigation. The impact of harvesting periods and geographical origins on the chemical composition of AM stems and leaves is not fully elucidated. Corresponding quality control standards also remain to be established.

Future research should prioritize comparative in vivo pharmacodynamic studies. These studies will compare AM stems and leaves with AM roots. The goal is to determine if above-ground parts can substitute for root materials. Furthermore, developing fingerprint chromatograms is essential. This helps standardize AM stem and leaf preparations. For novel bioactive compounds, integrated approaches should be used. These approaches include molecular docking simulations and animal experiments. The aim is to elucidate their mechanisms of action against metabolic disorders.

## 5. Pharmacokinetics Study

The active ingredients of AM have the potential for antioxidation and anti-inflammation. The absorption, distribution, metabolism, and excretion (ADME) of them after entering the body are the key to guiding clinical medication.

Rao et al. [173] established an LC-MS/MS method for quantifying formononetin concentrations in human plasma. Using this method, they compared the pharmacokinetic profiles of three AM formulations: total decoction pieces (TDP), ultra-pulverized powder (UP), and ultra-micronized granule preparation (UGP). The peak concentration (Cmax) and area under the plasma concentration–time curve (AUC) of UGP and UP were significantly higher than those of TDP, with the relative bioavailability of UGP and UP exceeding threefold that of TDP. This enhancement may be attributed to the disruption of plant cell walls by ultra-micronization technology, which improves the dissolution and absorption efficiency of formononetin. Formononetin undergoes enterohepatic circulation in vivo, and this phenomenon is most pronounced in the UGP group. This mechanism compensates for formononetin’s relatively short elimination half-life and prolongs its pharmacological effects. No significant difference in bioavailability was observed between UP and UGP. As a granular formulation, UGP avoids common storage challenges such as oxidation and moisture absorption. Pharmacokinetic data indicate that UGP exhibits stable absorption and minimal inter-individual variability, making it a more suitable candidate for clinical application as the preferred AM formulation [173].

Xiang et al. [174] proposed the concept of phenomenological pharmacokinetics (PhenoPK), which calculated comprehensive pharmacokinetic parameters by analyzing both the original components and metabolites of Chinese medicines in vivo. PhenoPK incorporates low-abundance constituents and metabolites, providing a simplified operational approach that reflects the holistic nature of herbal medicines. In rat plasma, 11 original components and 116 metabolites of AM were identified. Co-administration with *Saposhnikovia divaricata* (SR) enhanced the biphasic pharmacokinetic profile of AM. When administered alone, AM exhibited a peak time (P-Tmax) of 2.55 h and a mean residence time (P-MRT) of 10.71 h; following combination with SR, P-Tmax extended to 6.60 h and P-MRT increased to 14.75 h. This shift may be associated with SR-induced alterations in the metabolism of isoflavone components in the liver and intestine, resulting in a slower onset but prolonged duration of action [174]. PhenoPK is particularly suitable for studying complex Chinese medicine systems, as it provides a novel approach for investigating compatibility mechanisms and active substances. However, several limitations remain, including potential inaccuracies in peak area quantification for low-abundance components and reliance on prior metabolic studies. Since PhenoPK parameters are based on relative peak areas, they require validation using conventional quantitative methods.

Wang et al. [175] were the first to develop a quantitative analytical method for APSs in mouse plasma and tissues using reductive amination. They labeled APSs with the water-soluble near-infrared fluorescent probe IR783. After a single intravenous dose (30 mg·kg^−1^) in mice, Tmax was 0.67 ± 0.26 h, Cmax was 1599.29 ± 159.30 mg·L^−1^, and the elimination half-life (t_1/2_) was approximately 30 h. APSs were rapidly absorbed and slowly eliminated; additionally, enterohepatic circulation was observed, with a secondary absorption peak appearing at 6 h post-dosing. Following administration, APSs were predominantly distributed in the spleen, liver, kidney, lung, and heart, organs closely associated with immune and metabolic functions [175]. This study elucidated the in vivo disposition and pharmacological mechanisms of APSs, laying a foundation for their formulation development.

Du et al. [36] investigated the pharmacokinetic profiles of five major active constituents in rat plasma after oral administration of AM leaf extract at low (97.2 mg/kg), medium (324 mg/kg), and high (648 mg/kg) doses. These constituents—rhamnocitrin 3-glucoside, tiliroside, rhamnocitrin 3-neohesperidoside, huangqiyenin R, and huangqiyenin R (note: duplicate constituent name retained as per original text)—were rapidly absorbed (Tmax: 1.42–2.27 h) and eliminated at a moderate rate (t_1/2_: 5–9 h). Both Cmax and AUC showed dose-dependent increases [36]. These findings provide experimental support for pharmacological research, clinical application, and health product development involving AM leaves, facilitating their rational utilization.

Du et al. [176] explored the effects of T2DM status and AM co-administration on the pharmacokinetics of dapagliflozin (DAPA) in rats. In healthy rats, co-administration of AM increased DAPA’s Cmax and shortened its tmax; in contrast, in T2DM rats, co-administration reduced DAPA’s Cmax, prolonged its t_1/2_, and increased its apparent volume of distribution (V) [176]. These results suggest that AM enhances DAPA absorption and tissue distribution. Clinically, the diabetic status of patients should be considered when combining these two agents: in T2DM patients, the combination may extend DAPA’s duration of action, necessitating blood glucose monitoring to prevent hypoglycemia.

Zhao et al. [177] evaluated the effect of AsIV on the pharmacokinetics of febuxostat in rats with hyperuricemic nephropathy (HN). HN rats had renal dysfunction, and febuxostat accumulated in their bodies. Oral administration of AsIV reduced this accumulation. The effect was dose-dependent. However, intraperitoneal administration of AsIV had no significant effect. This difference suggests that AsIV works through the gut microbiota. Specifically, AsIV decreases the number of urease-producing bacterial genera. This inhibits the breakdown of urea into ammonia. It also suppresses the urea–ammonia hepatoenteric circulation. In turn, this reduces kidney damage caused by ammonia. Meanwhile, AsIV promotes the production of SCFAs. They help protect renal function. However, these findings need to be verified in human studies. Additionally, the molecular mechanisms behind the interaction between AsIV and gut microbiota are still unclear [177].

Yu et al. [26] improved the understanding of AM pharmacokinetics in non-rodent species. They did this by analyzing six major components in *beagle dog* plasma. The dogs had received an oral dose of AM water extract. The six components were calycosin-7-O-glucoside (CG), ononin, methylnissolin-3-O-glucoside (MG), formononetin, AsIV, and astragaloside II. Flavonoid glycosides were absorbed and eliminated quickly. This is probably because they have high polarity and are easily absorbed by the intestines. In contrast, saponin components were absorbed and eliminated more slowly. A possible reason is their large molecular size and low lipid solubility. These properties may prevent them from being absorbed by the intestines or metabolized by the liver. Formononetin had a higher AUC than ononin. This may be because ononin undergoes deglycosylation in the body to form formononetin [26]. This suggests that the in vivo activity of AM is also mediated by its metabolites.

Most existing research has focused on single components or total extracts. However, the intricate interactions among flavonoids, saponins, and polysaccharides are still largely unknown. Individuals with MetS often have pathological alterations. These alterations include IR and abnormal hepatic and renal functions. These factors can significantly affect the ADME processes of drugs.

Future studies should place greater emphasis on the application of PhenoPK. Integrating advanced technologies like metabolomics can help systematically analyze pharmacokinetic interactions. These interactions occur among multiple components of AM under coexisting conditions. The interaction between AsIV and APS needs to be clearly elucidated. Furthermore, animal models should be developed. These models should represent distinct pathological stages of MetS. They will help better simulate clinical scenarios. Additionally, investigating the underlying mechanisms is necessary. These mechanisms explain how pathological factors influence the ADME processes of AM constituents.

## 6. Safety Evaluation of AM

Pharmacokinetic studies have clarified the ADME characteristics of the components of AM in the body. Patients with MetS often need prolonged pharmacotherapy. Therefore, the long-term clinical safety of AM is of great importance for its therapeutic application.

Chan et al. [69] evaluated the safety of AM as an adjunctive therapy for patients with T2DM complicated by CKD. AM granules (equivalent to 15 g of crude drug) were administered for 48 weeks. This treatment did not significantly increase the risk of serious adverse events. Additionally, no statistically significant difference in the incidence of adverse events was found between the AM group and the standard care group. These results indicate that AM has an overall favorable safety profile [69].

Stępnik et al. [178] treated the human neuroblastoma cell line (SH-SY5Y) with AsIV. The concentrations of AsIV used were 6, 12.5, and 25 µg/mL. After 24 h of exposure, cell viability was assessed. No notable cytotoxicity was observed, even at the highest concentration tested. Moreover, there were no significant differences in cell viability between the AsIV-treated groups and the saline control group. In a parallel experiment, *zebrafish* embryos (1 h post-fertilization) were exposed to 25 µg/mL of AsIV. The exposure lasted for 96 h. No differences in mortality, hatching rate, or morphological abnormalities were detected between the AsIV-treated group and the control group. The tactile responses of the embryos also remained normal. These findings confirm that AsIV has a favorable safety profile: it shows no toxicity to neural cells in vitro and no adverse effects on embryonic development or function in vivo [178].

However, current safety assessments of AsIV have limitations. They mainly reflect short-term developmental toxicity. Importantly, there is a lack of in vivo data from mammalian models. Potential toxicity at higher concentrations has not been fully explored, and the observation period in existing studies was relatively short. Therefore, the long-term safety and efficacy of AsIV cannot be evaluated based on current research.

LS-102 is a novel derivative of AsIV. Wu et al. [179] investigated LS-102 using molecular dynamics simulations and secondary structure analysis. Their results showed that when LS-102 binds to human serum albumin (HSA), it does not cause significant changes to HSA’s secondary structure. This suggests that LS-102 does not damage HSA’s structural integrity or impair its function. Subsequently, *zebrafish* embryos were treated with different concentrations of LS-102. These experiments identified the maximum tolerated concentration (MTC) of LS-102 as 100 μg/mL. The data confirm that LS-102 has favorable acute safety when used at or below this concentration [179].

The current safety evaluation of AM features short-term, regular dosage and healthy models, which does not meet the clinical needs of MetS. There is still a lack of toxicity data for AM. This data refers to high-dose and long-term exposure to AM. Furthermore, safety assessments in vulnerable subpopulations are notably limited. These subpopulations include elderly individuals and MetS patients with hepatic or renal impairment. Additionally, research on AM’s safety profile is insufficient. This research focuses on AM when co-administered with commonly prescribed MetS medications. Conduct long-term, high-dose toxicity studies on AM and its active components. Monitor liver function, kidney function, and histopathological changes in mammalian models. These models include rats and dogs. Establish corresponding animal models for special populations. Use pregnant rats and rats with renal failure. Evaluate the safety of AM using these models.

## 7. Conclusions and Future Perspectives

Based on the above discussion, AM, a natural medicinal plant with considerable therapeutic potential, exerts key regulatory effects on MetS and associated diseases through multi-target mechanisms.

In recent years, newly identified compounds from AM have mainly included triterpenoid saponins. These may represent only a portion of its active constituents. Flavonoids and polysaccharides still possess considerable research value for further extraction and isolation. The structural elucidation and identification of novel compounds remain incomplete. Some newly discovered constituents, such as astragaloside A, exhibit high biological activity, yet their pharmacological effects and mechanisms of action are not fully understood. Further in-depth investigation is needed, which would support the medicinal development and utilization of AM. Chemical composition of AM varies significantly depending on its geographical origin and harvesting period. A standardized evaluation system based on chromatographic fingerprinting is required. Due to the limited availability of wild AM resources, exploring non-traditional plant parts such as stems and leaves may help alleviate resource pressure. This would also deepen our understanding of the whole plant, allowing for a comprehensive assessment of its medicinal value and enhancing its economic potential.

AM and its active ingredients demonstrate multiple pharmacological effects in the management of MetS and related disorders. Studies suggest that AM exerts regulatory effects on metabolic diseases such as T2DM, NAFLD, obesity, hypertension, and CVD through mechanisms including improvement of IR, protection of pancreatic β-cell function, modulation of gut microbiota, anti-inflammatory and antioxidant activities, and regulation of lipid metabolism. Despite promising results, current studies have limitations. Most findings are derived from in vitro experiments or animal models. Clinical evidence on safety and efficacy remains insufficient. Future research should prioritize large-scale randomized controlled trials to provide more reliable clinical evidence. Additionally, the synergistic interactions among multiple components in AM are not yet clearly defined. UPLC-MS/MS and in vitro intestinal absorption models combined with HPLC-PDA-MS have been successfully applied to the screening and identification of active components in AM. In the future, molecular docking technology can be further integrated to predict the target sites of new components. Metabolomics and 16S rRNA sequencing are suitable for investigating the interactions between intestinal flora and metabolism. Furthermore, proteomics and transcriptomics can be integrated to reveal the multi-target and multi-pathway metabolic regulatory effects of AM. Individual variations in gut microbiota may also influence the therapeutic outcomes of AM. Future studies should explore precise microbiota–host metabolic regulation mechanisms to support personalized medicine. It is also worth investigating the potential synergistic effects of AM in combination with conventional therapies, such as antidiabetic or statin medications. AM has achieved results in formulation optimization, pharmacokinetic characteristics, and short-term safety. However, the absorption and metabolic patterns of components under pathological conditions remain unclear. The pharmacokinetic interactions among multiple components have not been clarified. It is necessary to improve pharmacokinetic and safety data, explore the pharmacokinetic characteristics under pathological conditions, supplement long-term and special population safety evaluations, and clarify the risks of combined medication.

This review provides a reference for further studies on AM. Current research on AM remains predominantly confined to preclinical models, and the synergistic effects of its multiple bioactive components are not yet fully elucidated. Further efforts should focus on enhancing clinical translation and investigating the mechanistic interactions among multiple components, which will provide a more solid scientific foundation for its application in the treatment of MetS.

## Data Availability

No new data were created or analyzed in this study. Data sharing is not applicable to this article.
